# Mitochondrial DNA Diversity of Modern, Ancient and Wild Sheep *(Ovis gmelinii anatolica)* from Turkey: New Insights on the Evolutionary History of Sheep

**DOI:** 10.1371/journal.pone.0081952

**Published:** 2013-12-11

**Authors:** Sevgin Demirci, Evren Koban Baştanlar, Nihan Dilşad Dağtaş, Evangelia Pişkin, Atilla Engin, Füsun Özer, Eren Yüncü, Şükrü Anıl Doğan, İnci Togan

**Affiliations:** 1 The Department of Biological Sciences, Middle East Technical University, Ankara, Turkey; 2 Genetic Engineering and Biotechnology Institute, TUBITAK Marmara Research Center, Kocaeli, Turkey; 3 Department of Settlement Archaeology, Middle East Technical University, Ankara, Turkey; 4 Department of Archaeology, Cumhuriyet University, Sivas, Turkey; CSIRO, Australia

## Abstract

In the present study, to contribute to the understanding of the evolutionary history of sheep, the mitochondrial (mt) DNA polymorphisms occurring in modern Turkish native domestic (n = 628), modern wild (*Ovis gmelinii anatolica*) (n = 30) and ancient domestic sheep from Oylum Höyük in Kilis (n = 33) were examined comparatively with the accumulated data in the literature. The lengths (75 bp/76 bp) of the second and subsequent repeat units of the mtDNA control region (CR) sequences differentiated the five haplogroups (HPGs) observed in the domestic sheep into two genetic clusters as was already implied by other mtDNA markers: the first cluster being composed of HPGs A, B, D and the second cluster harboring HPGs C, E.

To manifest genetic relatedness between wild *Ovis gmelinii* and domestic sheep haplogroups, their partial cytochrome *B* sequences were examined together on a median-joining network. The two parallel but wider aforementioned clusters were observed also on the network of *Ovis gmelenii* individuals, within which domestic haplogroups were embedded. The *Ovis gmelinii* wilds of the present day appeared to be distributed on two partially overlapping geographic areas parallel to the genetic clusters that they belong to (the first cluster being in the western part of the overall distribution). Thus, the analyses suggested that the domestic sheep may be the products of two maternally distinct ancestral *Ovis gmelinii* populations.

Furthermore, *Ovis gmelinii anatolica* individuals exhibited a haplotype of HPG A (n = 22) and another haplotype (n = 8) from the second cluster which was not observed among the modern domestic sheep. HPG E, with the newly observed members (n = 11), showed signs of expansion. Studies of ancient and modern mtDNA suggest that HPG C frequency increased in the Southeast Anatolia from 6% to 22% some time after the beginning of the Hellenistic period, 500 years Before Common Era (BCE).

## Introduction

Archaeozoological evidence based on demographic parameters indicate that cattle, sheep, goats and pigs were domesticated in the region spanning from Central Anatolia to north of the Zagros Mountains [1]. In this center of animal domestication, sheep domestication started as early as 11,000 years before the present (BP). To further elucidate the evolutionary history of domestic sheep, the mtDNA control region (CR) and the cytochrome *B* gene (*cytB*) polymorphisms have been widely employed as is the case for many other livestock species [2]. Five haplogroups (HPG A to E) have been observed in sheep [3], [4], [5], [6], [7], [8], and all of them are present among the native sheep breeds of Turkey [8]. HPG B and HPG A, the most frequently observed haplogroups [8], and the third most common haplogroup, HPG C [9], were found to have undergone population expansions as one of the signs of domestication. HPG E and D did not have enough representatives; thus, the occurrence of expansion for these haplogroups could not be tested.

Wide scale geographic distribution patterns of endogenous retrovirus insertion site polymorphisms suggested that there were two major waves of sheep migrations: the spread of primitive domestic sheep, presumably from the center of animal domestication, was followed and replaced by the second wave of sheep migration from Southwest Asia with improved traits around 5000 years BP [10]. It would be interesting to have a closer look at the mtDNA haplogroup distribution in native sheep breeds of Turkey to unravel the imprints of major migrations from the maternal side of the sheep. In native sheep breeds of Turkey, although the polymorphisms of mtDNA CR [5], [8], [11], *cytB* [8] and even complete mtDNA sequences [12] have been examined, spatial mtDNA haplogroup distribution spanning the whole Turkey has not been studied yet.

Regarding wild sheep, the mtDNA sequences of *Ovis ammon* and *Ovis vignei* were highly deviant from the sequences of domestic sheep in the phylogenetic analysis [4], [7], [8], [12], [13]. Therefore, Asiatic mouflon (O*vis orientalis*), named *Ovis gmelinii* (*O*. *g*.) by recent nomenclature, [14], [15] was suggested as the ancestor of domestic sheep [4]. On the other hand, *O. g. musimon* and *O. g. ophion*, whose mtDNA CR belonged to HPG B [4], [11], were accepted as the ferals [4], [5], [8], [13], [16] of early domesticated sheep. The fragmented wild *O. gmelinii* populations are found in the area stretching from Central Anatolia (*O. g. anatolica*) to the Strait of Hormuz: Armenia and Northeast Iran (*O. g*. *gmelinii*), Esfahan (*O. g. isphahanica*), Laristan (*O. g. laristanica*) [17]. Genetic relatedness between the mtDNA of modern *O. gmelinii* individuals from these fragmented populations and domestic sheep mtDNA haplogroups have not been examined before. Such analysis may provide information about the history of sheep domestication.

*O. g. anatolica* is now represented by a single population at the Bozdağ protection area in Konya province of Central Anatolia. *O. g. anatolica* was proposed as the ancestor of HPG B [13]. However, the CR of the single available sample of *O. g. anatolica* belonged to HPG A [11]. For a few (n = 4) *O. g. anatolica* individuals, *cytB* sequences were determined, but not their relatedness to the haplogroups observed among domestic sheep [18], [19]. Thus, a larger number of *O. g. anatolica* individuals should be examined to understand if they could be the ancestors of HPG B of mtDNA lineage.

Although a pattern of genetic variation based on the samples of the current (modern) population is insightful regarding the possible evolutionary history of livestock [20], [21], [22], [23], [24], studies of ancient DNA (aDNA) may reveal valuable information about concealed population events such as human and animal migrations, extinctions or genetic bottlenecks. For example, the total replacement of a group of domestic pigs in Europe between 3900 -700 BCE, was revealed by analyzing aDNA [25].

In the present study, 628 modern samples from 13 native sheep breeds covering the whole of Turkey were examined with respect to their mtDNA haplogroup compositions to unravel the pattern of haplogroup distributions in search of the evolutionary history of domestic sheep in Turkey. The mitochondrial DNA CR of some of the representatives of the mtDNA haplogroups was sequenced to increase the number of available sequences in particular for the relatively rare mtDNA haplogroups: HPG C, HPG D and HPG E. When possible, they were subjected to tests of expansion. Furthermore, the mtDNA CR sequences of 30 Anatolian mouflon (*O. g. anatolica*) individuals (4 of them also for *cytB*) were sequenced and their genetic diversity relative to domestic individuals was identified. Moreover, the *cytB* sequences of *O. g. anatolica* and domestic sheep were examined comparatively with the sequences from other wild and feral *O. gmelinii* populations using the accumulated data from the literature [12], [18], [19], [26]. Finally, aDNA of sheep from an archaeological site Oylum Höyük in Kilis province, which is located in Southeastern Turkey [27], were examined to understand the temporal haplogroup changes in the region.

It is believed that the results of this study, samples being from or near to the center of animal domestication, will contribute to the understanding of sheep domestication history and to the history of domestic sheep of Turkey.

## Materials and Methods

### Ethics Statement

Modern sheep blood samples were collected with the approval of the Istanbul University Veterinary Faculty Ethics Committee (permit number: 2006/172).

*Ovis gmelinii anatolica* blood samples were collected with the approval of the Selçuk University Veterinary Faculty Ethics Committee (permit number: 2009/041) and were collected by the General Directorate of Nature Conservation and National Parks, Turkish Republic Ministry of Forestry and Hydraulic Works. The samples were studied with the permission of the institution (permit number: 797 dated 22/12/2009) and with the approval of the Local Committee on the Ethics of Animal Experiments of the Middle East Technical University (permit number: 2009/18).

The archaeological samples of the study were obtained from the repository of Oylum Höyük excavation with the permission of the Directorate of Gaziantep Museums (permit number: B.16.KVMG.4.27.00.01.152/995). The samples were studied with the approval of the Local Committee on the Ethics of Animal Experiments of the Middle East Technical University (permit number: 2011/07).

All necessary permits were obtained for the described study, which complied with all relevant regulations.

### Samples, sampling and DNA extraction

Blood samples (∼10 cc) were drawn by licensed veterinarian experts from the vena jugularis of 628 unrelated domestic sheep using K3 EDTA vacuum tubes. These domestic sheep represented thirteen native sheep breeds in Turkey: Karayaka (KRY), Akkaraman (AKK), Gökçeada (GOK), Dağlıç (DAG), Morkaraman (MRK), Kıvırcık (KIV), İvesi (IVE), Herik (HER), Karagül (KRG), Hemşin (HEM), Çineçaparı (CIC), Sakız (SAK), Norduz (NOR). Breeds, the provinces they were collected from, their tail types, sample sizes and coordinates for the centroids of sampling sites are listed in Table S1. Some breeds, such as Akkaraman, Kıvırcık and Çineçaparı were sampled from 2–3 flocks. For the other breeds on average 9.8 flocks per breed were sampled. The locations of these flocks and their sample sizes are given in Table S2.

Thirty blood samples were obtained from Anatolian mouflon (*O. g. anatolica*) in two different years (2009 and 2010) from three locations in the 42000 km^2^ Bozdağ protection area in Konya province of Central Anatolia. *O. g. anatolica* have gone through a bottleneck recently and the population declined to 15–20 individuals in the 1970s [28]. The size of the *O. g. anatolica* population is now nearly 500 in the Bozdağ region (personal communication).

Sheep samples were obtained from the archaeological excavation site of Oylum Höyük located near Kilis province in Southeastern Turkey. This site is one of the largest Bronze Age mounds in the region [27].

The ancient samples used in the study were obtained from the repository of the archaeological site (sample and archaeological codes are given in Table S6). The differentiation of sheep mandible and metapodia samples from those of goats was based on the criteria described in Supplementary Text S1. More information related to ancient samples and how they were studied can be found in Supplementary Text S2.

For the modern individuals, DNAs were isolated from blood samples according to the phenol-chloroform DNA extraction protocol [29]. The aDNA isolations were carried out following Rohland et al.'s [30] method in a physically isolated laboratory dedicated to ancient DNA analysis.

### Dating of aDNA Samples

The dating of ancient bones was in accordance with the chronology assigned to the stratigraphic layers and archaeological context by the director of the excavation. Bone samples OY019-2, OY042-2 and OY044-2 were dated with the Accelerator Mass Spectrometry (AMS) radiocarbon dating method at the Beta Analytic Inc. Laboratory (Miami, USA). Radiocarbon ages were calibrated using the IntCal09 curve [31].

### Haplogroup assignment and sequencing

Single Strand Conformational Polymorphism of the mtDNA ND2 region (ND2-SSCP) was used to assign haplogroups to 628 domestic sheep. The details and the validity of the method were described in Yüncü et al. [32]. Among the 628 samples, the mtDNA CR of 240 sheep was sequenced as detailed in Yüncü et al. [32]. The length of employed CR sequences was 1180 bp long and corresponded to positions 15437–16616 on the reference sequence (AF010406) [33].

For ten domestic samples and four *O. g. anatolica* individuals, the 1272 bp fragment of *cytB* region (AF010406 positions 14078–15349) was amplified and sequenced by the protocol given in Meadows et al. [34]. After multiple sequence alignment, the final length of sequences was 1042 bp long, which corresponded to positions 14180–15221 on the reference sequence (AF010406).

The 144 bp fragment of mtDNA encompassing partial tRNA^Pro^-CR (AF010406 positions 15391–15534) was sequenced for 33 ancient samples by using primers from Cai et al.'s [35] study since this region was observed to be useful in identifying all 5 mtDNA haplogroups (A to E) as seen in Table S3. The mitochondrial DNA haplogroups were determined based on different nucleotide positions as indicated in Table S3 with respect to the reference (AF010406) sequence.

### Data Analyses

For the modern samples (domestic and *O. g. anatolica*), the lengths (75 bp/76 bp) of the repeat unit in CR (starting from the 15640^th^ nucleotide of AF010406) as well as the number of repeat units for each sequence (n = 240) were examined.

A neighbor-joining tree based on mtDNA CR sequences of the modern samples was constructed by MEGA 5 [36]. The complete tandem repeat region was excluded from CR sequences for the tree construction. The Tamura-Nei model [37] was applied to the sequences with 1000 bootstrap value. As out-groups *O. vignei* (AY091490, AY091491) and *O. ammon* (AF242347, AF242348) sequences [13] and as a reference for each haplogroup HM236174–83 sequences [12] were used.

For the modern samples, nucleotide diversity (π) and haplotype diversity for each haplogroup, as well as the average number of nucleotide differences per site (Dxy) between haplogroups were calculated by DnaSP v.5 [38]. For the calculation of Dxy between *O. g. musimon* and HPG B, *O. g. musimon* samples HM236184–85 [12], AY091487–88 [13], AF039579 [4] from previous studies were used.

Fu's *F_S_* [39] test, Tajima's *D* [40] test and mismatch distributions [41] were calculated by Arlequin 3.11 [42] for each haplogroup, except HPG D since it had a small sample size. The validity of the sudden population expansion model [43] was tested using the sum of square deviations between the observed and expected mismatch values [44]. For the rare HPG E, the analyses were repeated for three cases; E1: sequences (n = 11) were in 886 bp length and were the samples of the present study; E2: sequences (n = 15) were in 886 bp length and two sequences (HM236182–83) from Meadows et al. [12] and another two sequences (AY829385 and AY829404) from Guo et al. [6] were added to the sequences of E1; E3: sequences (n = 20) were in 665 bp length and DQ097468 [5], HM042760–61, HM042785, HM042838 sequences were added to the sequences of E2. The sequences used in neighbor-joining tree construction, genetic diversity calculations and mismatch distribution analyses are summarized in Table S5.

The haplogroup frequencies obtained as a result of ND2-SSCP analysis (n = 628) were used to construct the pie charts to demonstrate the spatial distribution of haplogroups across Turkey. The haplogroup frequencies of *O. g. anatolica* and ancient samples based on mtDNA CR sequences are also given as pie charts.

To examine the spatial distribution of haplogroups among the modern domestic sheep of Turkey, spatial autocorrelation coefficients (r) [45] at each of 150 km distance classes based on the flock information of the samples (n = 628) were used (Table S2). The distance class was chosen under the assumption that it is the diameter of a circle which contains the sample collection sites for a breed. The correlogram was constructed with GENALEX 6 [46]. The 95% CI belt for the spatial autocorrelation coefficients (r) under the null hypothesis of no genetic structure among the samples over the space was determined by 999 permutations. The 95% CI of r for each distance class was determined using 1000 bootstrap replicates.

For the modern samples (domestic, wild and feral sheep), a Median-joining (MJ) network with partial mtDNA *cytB* region was constructed by NETWORK 4.6.1.0, nucleotide positions were weighted as was done by Olivieri et al. [47]. The sequences retrieved for MJ network construction are listed in Table S4.

The sequences of mtDNA CR of domestic sheep (n = 240), the mtDNA CR of *O. g. anatolica* (n = 30), the partial *cytB* of domestic sheep consisting of 2 from each haplogroup (n = 10) and the partial *cytB* of *O. g. anatolica* consisting of 2 from each haplotype (n = 4) that were obtained in the present study are available in GenBank accession no. KF677024–KF677307.

## Results

### Analyses of mtDNA CR

#### Modern domestic sheep and *O. g. anatolica*

**Figure 1:**
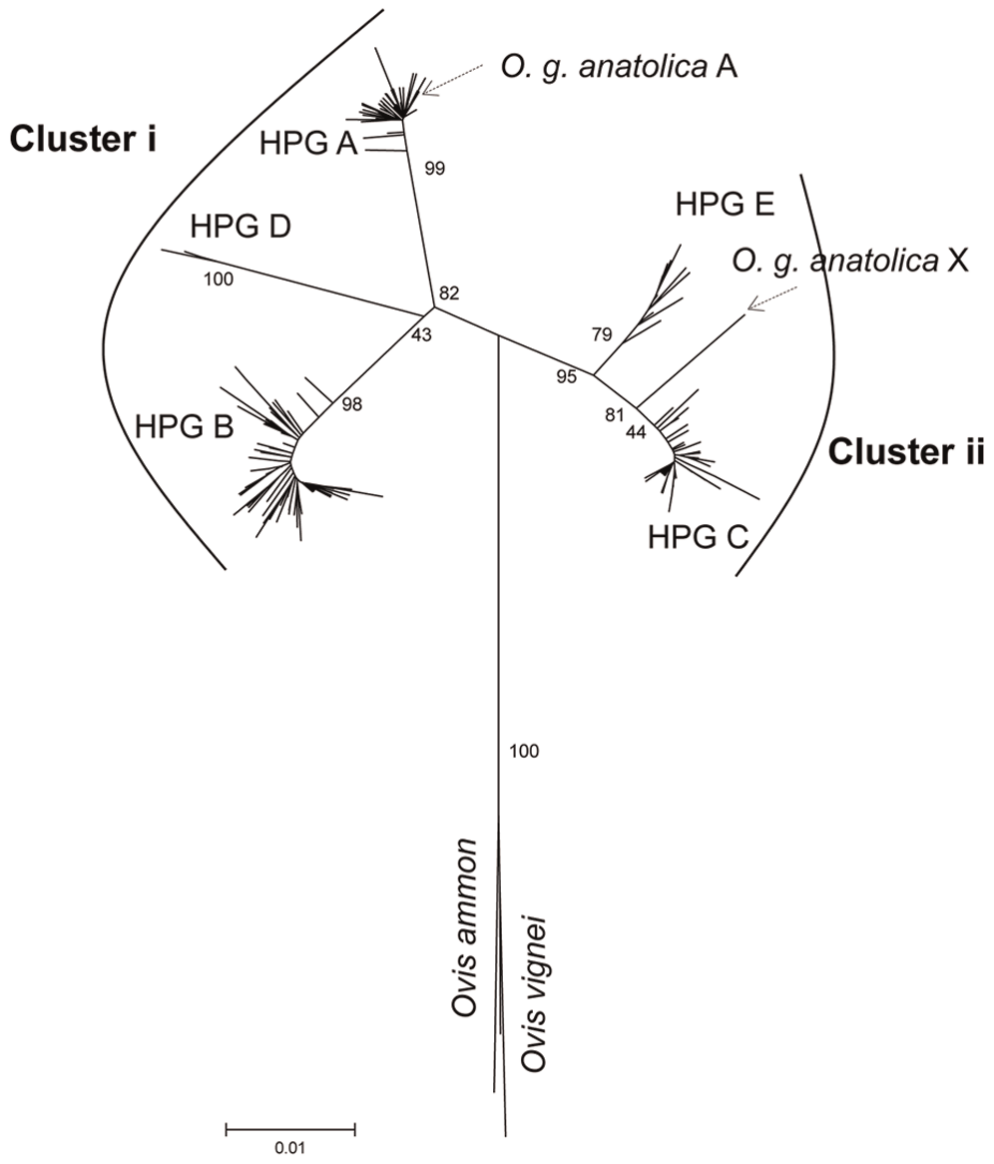
The neighbor-joining tree of mtDNA CR sequences from domestic sheep and *O. g. anatolica* samples. The haplogroups (HPGs A–E) and two clusters (Cluster i and Cluster ii) formed by the sequences are designated. The bootstrap values are indicated on the main branches of the tree.

The mtDNA CR sequences (n = 240) of domestic sheep representing HPG A (n = 70), B (n = 88), C (n = 69), D (n = 2), and E (n = 11) were used to construct the neighbor-joining tree (Figure 1) together with the sequences of *O. g. anatolica* and the reference sequences as described in Table S5. *O. g. anatolica* sequences (n = 30) provided two haplotypes: the first one (n = 22) clustered with HPG A and was named *O. g. anatolica* A. The second haplotype (n = 8), which hereafter will be referred to as *O. g. anatolica* X, was placed in between the HPG E and HPG C sequences on the neighbor-joining tree.

*O. g. anatolica* X had not been observed previously among the domestic sheep studied, and on the neighbor joining tree the branch corresponding to this haplotype was supported by a high bootstrap value of 81% (Figure 1). Thus, it is quite distinct from all of the haplogroups observed in domestic sheep, even from the two (HPG E and HPG C) with which it shares a branch.

The sequence diversity among the sheep of the present study was firstly assessed by considering the length of the repeat unit, which generally starts at the 213^th^ base from the 5′ end of the mtDNA CR (15650^th^ base on AF010406) sequences. The length of the first repeat unit was always 75 bp. Interestingly, in the following units, the 76 bp length was exclusively associated with HPG C, HPG E and *O. g. anatolica* X, whereas the 75 bp repeat unit was always observed with HPGs A (including *O. g. anatolica* A), B and D in the employed sequences of Figure 1. Thus, the bifurcation from the node where the out groups (*Ovis ammon and Ovis vignei*) join the neighbor-joining tree; thus, the formation of Cluster i and Cluster ii (Figure 1) is also marked by the existence of two different (75 bp/76 bp) repeat lengths in the second and subsequent repeat units of the mtDNA CR. This observation will be referred to as two different (75 bp/76 bp) repeat lengths in the rest of the text. The observed number of repeat units ranged between 3 and 5. The most frequent repeat unit number was four (256/279). Five repeats were observed among the samples with HPG C (n = 3) and in one of the *O. g. anatolica* X.

The genetic diversity in terms of number of haplotypes, haplotype diversity and nucleotide diversity for each haplogroup based on their CR sequences is presented in Table 1. In this analysis, *O. g. anatolica* haplotypes were not considered since *O. g. anatolica* is composed of only two distinct haplotypes (A and X). Haplogroups A, B and C, were represented by relatively high haplotype numbers (n≥43). HPG B, represented by the highest number of sequences, exhibited the highest number of haplotypes. HPG E and HPG D were represented by 11 and 2 sequences, respectively; however, they revealed the highest nucleotide diversities. The least haplotype and nucleotide diversity was exhibited by HPG C.

**Table 1:** Summary of mtDNA haplogroup diversity of domestic sheep.

	HPG A	HPG B	HPG C	HPG D	HPG E
**Number of sequences**	70	88	69	2	11
**Number of haplotypes**	48	66	43	2	9
**Haplotype diversity**	0.975±0.011	0.987±0.006	0.947±0.020	1.000±0.500	0.964±0.051
**Nucleotide diversity (x10^−3^) (π)**	3.9±0.29	5.5±0.32	2.9±0.26	5.7±2.84	6.5±0.94

The average number of nucleotide differences per site (Dxy) between the haplogroups and *O. g. anatolica* haplotypes, as well as the HPG B of *O. g. musimon*, are depicted in Table S7. *O. g. anatolica* X seemed to be considerably closer to C (Dxy: 0.0149) than to E (Dxy: 0.0229). The feral remnants of HPG B, *O. g. musimon* diverged from HPG B (Dxy: 0.0075) by half of the divergence between *O. g. anatolica* X and its closest haplogroup: HPG C (Dxy: 0.0149). *O. g. anatolica* A was very similar to HPG A (Dxy: 0.0032) and the divergence is half of that between *O. g. musimon* and HPG B (Dxy: 0.0075).

Each group of samples in HPG A, B and C showed bell-shaped mismatch distributions (not shown) as had been observed previously [8], [9]. In the present study, 11 new samples of HPG E were observed. Then for HPG E, mismatch distributions employing three different sample sizes (11, 15 and 20) and sequence lengths (partial and complete CR) were obtained and given in Figure S1. The observed mismatch distributions supported the sudden population expansion model [43] according to calculated p(E1): 0.26, p(E2): 0.77 and p(E3): 0.60 values of the sum of squared deviations test [44]. The mean mismatch values were parallel to the within group nucleotide diversities (Table S8). Fu's F_S_ statistics and Tajima's *D* statistics showed significant deviations from neutrality in HPG A–C and in HPG E1–E3 (Table S8).

#### Ancient sheep

From 57 ancient sheep samples of Oylum Höyük, the aDNA of more than half (24 teeth samples from mandible and 9 metapodia samples) could be isolated. The DNA obtained from the first extraction was amplified twice for most of the (31/33) samples as shown in Table 2. The DNAs of some samples (n = 3) were extracted twice. Thus, at least two sequences per sample were obtained for most of the (32/33) ancient samples. Negative controls were performed on all of the extractions and PCR amplifications. Then samples were assigned to haplogroups (Table 2) using the reference nucleotide positions shown in Table S3. Although there were variations between the sequences of the same samples (not shown), these were not on the haplogroup identification sites. Therefore, the haplogroups of 32 samples were confirmed at least twice. Furthermore, the validity of haplogroup determination sites given in Table S3 was confirmed using 240 domestic samples from the present study.

**Table 2:** Summary of aDNA analyses.

Sample code (Number of DNA isolations/Number of PCR amplifications)	mtDNA CR positions (AF010406)					Haplogroup	Dating
	15459	15476	15484	15509	15512		
OY003-1 (2/4)	T	**.**	A	**.**	**.**	A	1800−1700 BC
OY018-1 (1/2)	T	**.**	A	**.**	**.**	A	1800−1700 BC
OY020-1 (1/2)	T	**.**	A	**.**	**.**	A	1800−1700 BC
OY024-1 (1/2)	T	**.**	A	**.**	**.**	A	1800−1700 BC
OY025-1 (1/2)	T	**.**	A	**.**	**.**	A	1800−1700 BC
OY065-2 (1/2)	T	**.**	A	**.**	**.**	A	1800−1700 BC
OY010-1 (2/4)	**.**	**.**	**.**	**.**	**.**	B	1800−1700 BC
OY042-2 (1/2)	**.**	**.**	**.**	**.**	**.**	B	1800−1700 BC
							(1880−1690 BC)*
OY027-1 (1/1)	T	**.**	A	**.**	**.**	A	1800−1600 BC
OY134-2 (1/2)	**.**	**.**	**.**	**.**	**.**	B	1800−1600 BC
OY086-2 (1/2)	**.**	**.**	**.**	**.**	**.**	B	1800−1600 BC
OY130-2 (1/2)	**.**	**.**	**.**	**.**	**.**	B	1800−1600 BC
OY044-2 (1/2)	**.**	**.**	**.**	G	**.**	C	1800−1600 BC
							(1880−1680 BC)*
OY133-2 (1/2)	T	**.**	A	**.**	**.**	A	1600−1200 BC
OY105-2 (1/2)	**.**	**.**	**.**	**.**	**.**	B	1600−1200 BC
OY067-2 (1/2)	**.**	**.**	**.**	**.**	**.**	B	1600−1200 BC
OY070-2 (1/2)	T	**.**	A	**.**	**.**	A	1200−900 BC
OY061-2 (1/2)	T	**.**	A	**.**	**.**	A	1200−900 BC
OY059-2 (1/2)	**.**	C	**.**	G	**.**	E	1200−900 BC
OY090-2 (2/2)	T	**.**	A	**.**	**.**	A	1200−330 BC
OY123-2 (1/2)	**.**	**.**	**.**	**.**	**.**	B	1200−330 BC
OY072-2 (1/2)	**.**	**.**	**.**	**.**	**.**	B	1200−330 BC
OY019-2 (1/2)	**.**	**.**	**.**	G	**.**	C	900−700 BC
							(1000−840 BC)*
OY078-2 (1/2)	T	**.**	A	**.**	**.**	A	700−330 BC
OY082-2 (1/2)	T	**.**	A	**.**	**.**	A	700−330 BC
OY110-2 (1/2)	T	**.**	A	**.**	**.**	A	700−330 BC
OY025-2 (1/2)	T	**.**	A	**.**	**.**	A	700−330 BC
OY142-2 (1/2)	T	T	A	A	T	A	700−330 BC
OY089-2 (1/2)	**.**	**.**	**.**	**.**	**.**	B	700−330 BC
OY091-2 (1/2)	**.**	**.**	**.**	**.**	**.**	B	700−330 BC
OY081-2 (1/2)	**.**	**.**	**.**	**.**	**.**	B	700−330 BC
OY138-2 (1/2)	**.**	**.**	**.**	**.**	**.**	B	700−330 BC
OY021-2 (1/2)	**.**	**.**	**.**	**.**	**.**	B	330−30 BC

* Dates by AMS 14C radiocarbon dating; Stands for the identical nucleotides. Dates by archaeological context;

The sigma calibrated age ranges, for AMS 14C radiocarbon dated samples OY019-2, OY042-2 and OY044-2 are 1000-840 BCE, 1880-1690 BCE and 1880-1680 BCE, respectively. The dates assigned by archaeological context and AMS 14C radiocarbon dating are in good agreement, as can be seen in Table 2. In this study, the dates 1800 BCE and 30 BCE were used as the earliest and the most recent dates, respectively, as determined by archaeological context. However, the majority of the samples in the present study belong to the 1800 BCE-330 BCE time interval which corresponds to a period within Bronze and Hellenistic ages.

For the ancient samples, the frequencies of haplogroups were calculated from the data given in Table 2.

Ancient samples (n = 33) spanning the period 1800−30 BCE exhibited high frequencies of HPG A (16/33, 48.5%), HPG B (14/33, 42.4%) and a low frequency of HPG E (1/33, 3%) as is the case today in Turkey (Table 2). During the second half of the Bronze age (1800−1200 BCE) and in the Iron and the beginning of Hellenistic ages (1200 BCE−330 BCE) frequencies of HPG A, HPG B and HPG C (For HPG A: 8/16 and 8/16 respectively, for HPG B: 7/16 and 6/16 respectively and for HPG C: 1/16 and 1/16 respectively) did not change. The aDNA for both HPG A and B frequencies were similar to present day frequencies observed in the region (in IVE 37% and 41%, respectively). However, among ancient samples from Oylum Höyük considerably less frequent HPG C (2/33, 6.1%) (Table 2) was observed relative to their modern counterpart of the region (IVE 22%) and compared to its close neighbourhoods (NOR 24%, AKK 14%) (Table S1).

### Spatial analysis of mtDNA CR haplogroups

**Figure 2:**
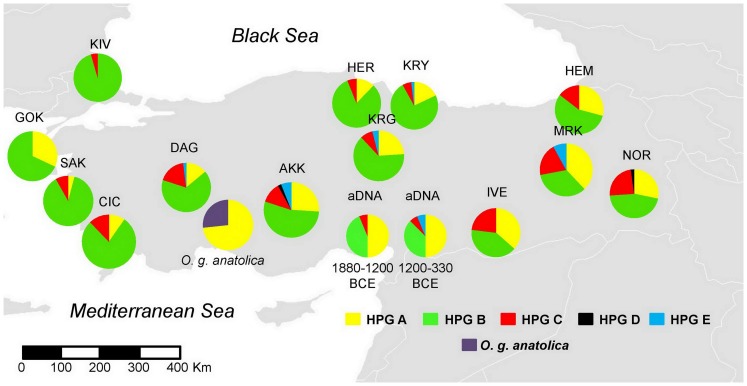
Haplogroup/haplotype compositions of the breeds, aDNA and *O. g. anatolica* on the map of Turkey. The locations of the pie charts on the centroids of the collection sites are all within the native distributions of the breeds or within the current day distribution for *O. g. anatolica*. Ancient DNA (aDNA) samples are located in the Kilis province, where Oylum Höyük is located. aDNA samples are considered in two successive time intervals therefore they are represented by two pie charts. The abbreviations of the breed names are given in the Materials and Methods section.

For the spatial pattern analysis, the HPG frequencies of breeds (Table S1) in the form of pie charts are shown on the centroids of the collection sites on the map of Turkey (Figure 2). The map shows the gradual change of haplogroup frequencies across Turkey from high frequencies of HPG B in the west and high frequencies of HPG A and C in the east, especially in Southeast Anatolia (Figure 2).

The existence of a spatial pattern of haplogroup distribution for the modern samples, as observed in the pie charts (Figure 2), was tested by spatial pattern analysis [48]. The spatial autocorrelation correlogram showed a non-random spatial distribution of haplogroup composition in Turkey (Figure S2).Of the 14 autocorrelation coefficients belonging to different distance classes, 11 were significantly (p≤0.001) different from the spatially random distribution. In general, there is a decrease in mtDNA haplogroup frequency resemblances (r) as a function of distance. However, the presence of a peak in the middle of the correlogram suggests that there may be an “intrusion” [49] of sheep with a distinct mtDNA haplogroup composition into Turkey.

### Analyses of mtDNA *cytB*

#### Phylogenetic relationship of modern *O. gmelinii* populations and domestic sheep haplogroups

**Figure 3:**
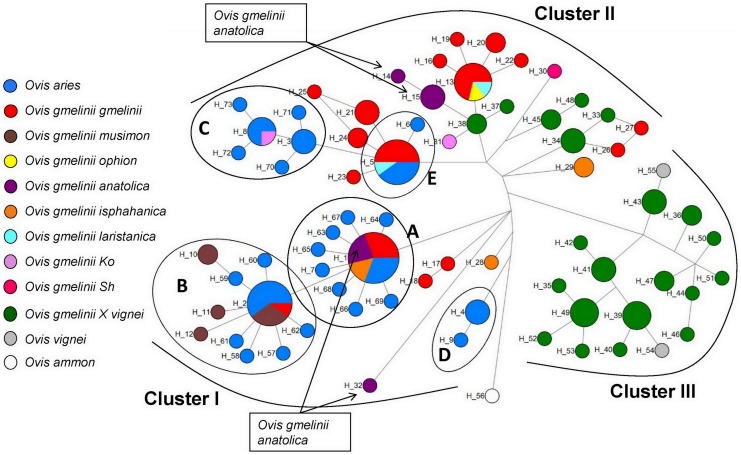
Median-joining network of mtDNA partial *cytB* sequences found within modern domestic and wild sheep. Nodes representing the haplotypes are proportional to the sample sizes used for the construction of the network. Haplotype names are given near the nodes. The ellipses labeled with letters A–E refer to the haplotypes of the individuals whose haplogroups were identified in domestic sheep in accordance with their CR sequences. The accession numbers of sequences used in the MJ network and their respective haplotypes are given together with the reference studies in Table S4.

The partial *cytB* region sequences of 10 domestic sheep (each haplogroup was represented by two individuals) and four individuals of *O. g. anatolica* (two for each observed mtDNA CR haplotype) of the present study were examined along with similar sequences from modern populations of wild sheep. The wild sheep represented a wide range of *O. gmelinii* populations including *O. g. anatolica* from Bozdağ [18], *O. vignei* and their hybrids *(O. gmelinii X O. vignei)* [19]. These samples, together with references from all of the haplogroups of domestic sheep (n = 33) [5], [12], *O. g. ophion* [26] and *O. g. musimon* [12], [19], [26] were employed to construct a median-joining (MJ) network (Figure 3). Information about the retrieved *cytB* reference sequences is presented in Table S4.

On the MJ network (Figure 3), where the *O. ammon* was the out-group, three distinct clusters of haplotypes were identified and indicated as Clusters I–III. Partial *cytB* sequences of the representatives of domestic haplogroups: HPG A, HPG B, and HPG D were in Cluster I. Two of the *O. g. anatolica* A samples from the present study exhibited the same haplotype referred to as H1 on the MJ network and it was in Cluster I. Previously, it had also been observed in *O. g. anatolica* by Rezaei et al. [19]. As it can be seen from Figure 3, H1 is one of the haplotypes of HPG A that can be observed in both wild and domestic sheep. The *O. g. musimon* samples (n = 7) (H2, H10–12) [12], [19], [26] and another *O. g. anatolica* sample (H32) studied previously [18] were in Cluster I as well. Partial *cytB* sequences of HPG C and E were in Cluster II. The two individuals exhibiting *O. g. anatolica* X haplotype revealed H15 haplotype on the basis of *cytB* sequences. H15 was in Cluster II. Thus, *cytB* based Clusters I and II are parallel to CR based Clusters i and ii. Previously, haplotype H14 (one mutation different than H15) as well as H15 were observed among the samples of *O. g. anatolica* [19]. The *O. g. ophion* (H13) from Cyprus, is also in Cluster II, is 2–3 mutations different from two of the *O. g. anatolica* haplotypes. Cluster II harbors some of the hybrids *(O. gmelinii X O. vignei)*, suggesting that for these hybrids the female parent belonged to *O. gmelinii*. The third cluster (Cluster III) is composed of *O. vignei*, and the hybrids, where presumably the females of these hybrids belonged to *O. vignei*. Figure 3 seems to differentiate the hybrids with respect to their maternal parents: If the mother was *O. gmelinii* it is in Cluster II; otherwise, it is a member of Cluster III. With respect to the genetic diversities of the first two clusters: there are a higher number of haplotypes observed among the *O. gmelinii* wilds, excluding hybrids and ferals (*O. g. musimon* and *O. g. ophion*), of Cluster II (n = 18) with haplotype diversity 0.934±0.023 compared to those of Cluster I (n = 12) with haplotype diversity: 0.604±0.150. These results show the presence of two clusters each with multiple and diverse mtDNA haplogroups in *O. gmelinii*.

### Phylogeography of modern *O. gmelinii*

**Figure 4:**
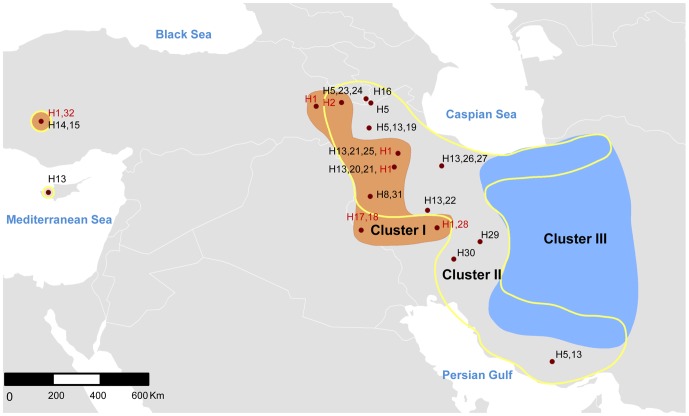
The distribution of wild samples that were employed in Figure 3. The collection sites for members of Clusters I, II and III are indicated on the map with the brown region, the region with yellow borders and the blue region, respectively. The exact locations of collection sites (solid red circles) together with haplotypes (as depicted in Figure 3) of the wild *O. gmelinii* members of Cluster I (typed in red) and Cluster II (typed in black) including *O. g. ophion* are shown. The exact locations of the hybrids (not shown) were used in drawing the borders of the region with yellow borders and the blue region. The map was created using ArcMap™ within ArcGIS Desktop 10.

The collection sites of wild (wild *O. gmelinii* ([18], [19], present study) the hybrids [19]) and *O. g. ophion* [26] individuals used in Figure 3 are shown in Figure 4. On the map (Figure 4), the collection sites of the members of the Clusters I, II and III were considered separately and they were delineated by the manually drawn borders. Thus the regions: the brown region, the region with yellow borders and the blue region in Figure 4, harbors the locations of the cluster members for the Clusters I, II, III respectively. Although they were not shown on the map, data points belonging to the hybrids were taken into account to construct the borders of Cluster II and III. In drawing the borders, the aim was to ensure the integrity of the region. For instance, when drawing the southwest limit of the region with yellow borders, a continuous area between the locations of H30 and H5,13 was assumed. The brown region, which lies in the western part of the present day distribution of the *O. gmelinii* wilds, represents the collection sites of wild individuals that have the same or similar genetic identity to those domestic sheep from HPG A, HPG B and HPG D. There is a single wild individual with H2 haplotype (HPG B) in the brown region to the northwest of the overall distribution of wild sheep (Figure 4), whereas the H1 haplotype (HPG A) seems to be widely distributed in the brown region. The *O. gmelinii* individuals related to haplogroups C and E of the domestic sheep are in the yellow-bordered region. H5 (HPG E) and H13 (observed in *O. g. ophion*) haplotypes are widely seen in the yellow bordered region. Finally, the hybrids whose mothers are presumably *O. vignei* (hybrids in Cluster III) are in the blue region.

On the basis of all its c*ytB* sequences (n = 8) ([18], [19], present study), *O. g. anatolica* seems to have members related to individuals in the brown region with H1 (n = 3) and H32 (n = 1) and the yellow bordered region with H14 (n = 1) and H15 (n = 3) (Figure 3, Figure 4). Currently, at two instances, H1 individuals are observed in the vicinity of H13 (also seen in *O. g. ophion*), the haplotype genetically close to H14 and H15 of *O. g. anatolica* (Figure 4).

## Discussion

The existence of five haplogroups for the mtDNA of domestic sheep has been well documented [8], [12], [47]. Genetic relatedness of these haplogroups, [8], [12] indicated that HPGs A, B, D and HPGs C, E form two groups of haplogroups. These groups were depicted as Clusters i and ii with respect to their mtDNA CR sequences in the present study (Figure 1).

Diversity in the sequence length of the repeat units in mtDNA CR (75 bp/76 bp) was previously reported by Meadows et al. [12]. The results of the present study revealed that the sequence length of the second or subsequent repeat unit in mtDNA CR serves as a marker to discriminate two clusters of haplogroups: HPGs A, B, D (75 bp) and HPGs C, E (76 bp). The latter cluster also included *O. g. anatolica* X. The generality of the observation was further confirmed by examining the previously published mtDNA CR sequences of domestic sheep (n = 683) covering the repeat region [3], [4], [6], [7], [12], [13], [33], [50].

The mtDNA CR sequences of wild *O. gmelinii* individuals have not been reported previously. However, considerable mtDNA *cytB* sequence data spanning individuals of a wide range of *O. gmelinii* populations were available in the literature [12], [18], [19], [26]. In Figure 3, partial *cytB* sequences of modern wild *O. gmelinii* individuals ([18], [19], present study), *O. gmelinii* hybrids [19], *O. g. ophion* [26] and *O. g. musimon* [12], [19], [26] were considered together with the samples of domestic sheep with known haplogroups. Then, the genetic relatedness of wild sheep to domestic sheep representing different haplogroups could be examined on the basis of their *cytB* sequences. The three clusters were observed in Figure 3 where two of them (Cluster I and Cluster II) covered all of the haplogroups observed in the domestic sheep and they corresponded to haplogroup Clusters i and ii, as was expected for the two regions of non recombining DNA molecule; mtDNA, but displaying higher genetic diversity within the clusters now. The presence of these two genetically distinct clusters prompted the proposition that the ancestors of these two mtDNA clusters may have had different geographic origins. It is known that wild animals or animals in their early domestication stages have been transported by hunter-gatherers over long distances or even by sea, for instance from the mainland to Cyprus as early as around 12,000 years BP [1]. Similarly, wild goats were thought to have been transported by humans, for example from the Central Iranian Plateau to Northern, Eastern and Central Anatolia before or in the early stages of the goat domestication [21]. Furthermore, it must be remembered that the modern distribution of wild *O. gmelinii* does not represent the distribution that existed before the Neolithic era [51], [52]. Despite these disturbances on the native distribution of wild sheep, the partitioned distribution of the geographic sites of modern wild sheep (Figure 4) parallel to their genetic distinctness (Figure 1, Figure 3) seemed to support the proposition. Thus, genetic and phylogeographic analyses indicated that the domestic sheep may be the products of two maternally distinct ancestral *Ovis gmelinii* populations. This hypothesis built on the basis of single locus of the sheep genome namely mtDNA and it needs to be confirmed by nuclear markers.

It is highly likely that the lands in Anatolia were the area harboring mainly the members of Cluster I (Figure 3) (because they were predicted to be on the western part of the *O. gmelinii*, distribution). The observance of relatively few wilds and considerably less haplotype diversity among the wilds in Cluster I compared to Cluster II, even when the hybrids were not considered, can be explained by the loss of diversity as a consequence of the retreat in natural distribution of *O. gmelinii* [51], [52]. This retreat could be due, at least partly, to the intrusion and the competition of domestic sheep in the former habitats of wild sheep [51].

Whether *O. g. anatolica* represents the part of the gene pool which had been in the area even before the Neolithic era cannot be answered currently. Unlike European mouflon, since *O. g. anatolica* harbored at least 3 haplotypes - H14 (not in the present study), H15 and H32 (not in the present study) - which were not observed in the domestic sheep it can be said that it is not totally composed of the feralized domestic sheep. The modern *O. g. anatolica* gene pool, being composed of H1 (HPG A) and H15, seems to be different from the gene pool of *O. g. ophion*, which in turn is composed of HPG B [11] and H13 [26].

The haplotype depicted as H32 of Cluster I, represented by an individual of *O. g. anatolica* sampled before 1999 (personal communication) from the distribution area of *O. g. anatolica* [18], seems to be extinct since it was not observed in the present study. Similarly, H14 of Cluster II [19] was not observed in the present study. It seems that, despite the current population size (nearly 500), genetic erosion has been continuing over the last decades. The absence of HPG B in *O. g. anatolica*, contrary to expectations [13], may indicate loss of this haplogroup during the fragmentation and bottleneck(s) upon losing its connection with the brown region (Figure 4) after geographic isolation around 6000 years BP [53]. However, during the bottleneck(s), the rare alleles are expected to be readily lost. Hence, even if it were in the *O. g. anatolica*, HPG B may have been rare.

Among the primitive sheep such as *O. g. ophion* (Cyprus mouflon), Soay sheep and Icelandic sheep exhibiting retrovirus insertion polymorphisms of the first sheep migration after domestication [10] seem to possess mtDNA HPG B [11] in abundance. HPG B is seen in Europe, Asia and Africa [7], [11], [54] as the most common HPG. However, the HPG B of domestic sheep was observed in a single individual (H2) among the samples of modern wild *Ovis gmelinii* (n = 48) [19]. Whether this observation is due to drift in the frequency of HPG B in wild sheep needs to be examined.

Members of HPG D were rare (2 out of 628) in the present study. It was previously observed [7], [8] very rarely (1/406, 2/197, respectively). The HPG D distribution seemed to be confined to eastern Anatolia and the Caucasus [7]. HPG D related individuals were not observed in wilds. Whether or not modern domestic individuals of HPG D correspond to an early local domestication requires further studies.

In the present study, HPG E was represented by 11 modern domestic sheep individuals (out of the 628 examined) and it was observed in Central, Eastern and Southeastern Anatolia but not in West and North Anatolia, except in KRY (Figure 2). HPG E was first observed in Turkey [5] and its few representatives extend to China [6] and Israel [8], [12]. Among the ancient samples of Oylum Höyük, it was again rare (1/33) (Table 2). In the present study, HPG E (just like HPGs A, B and C) showed signs of significant expansion. However, with the available data it can not be stated whether the expansion is associated with domestication.

For the breeds in Turkey, although they are not isolated, nonrandom distribution pattern of mtDNA haplogroup frequencies was observed (Figure 2, Figure S2). Eastern-Central Anatolian sheep (MRK, AKK, IVE, NOR, HEM) are mainly fat tailed sheep. They seemed to be different than the Western - Northwestern thin/semi-fat tailed sheep (KIV, GOK, KRY, SAK) with respect to their mtDNA haplogroup compositions (Figure 2). Non random pattern with the possible “intrusion” in the spatial autocorrelation analysis (Figure S2) might be due to the haplogroup composition differences of these two groups. Similarly, NOR of the Eastern Anatolia was observed to be different than SAK of Western Anatolia on the basis of SNP analysis [24] perhaps because of the differences in their origins. Among the ancient domestic sheep from Oylum Höyük, the HPG C frequency (6.1%) seemed to be lower at least until about 330 BCE, in the Hellenistic period, than what can be seen in the same region (Ivesi, Turkish Awasi, 21.6%) or around the region (Norduz 23.9%, Akkaraman 14%) today (Table S1). This observation can be explained by the genetic drift causing random changes in haplogroup frequencies in the recent generations and/or by the migration of HPG C rich sheep (at least to Southeast Anatolia) after 330 BCE.

In this study, a closer look was given at modern domestic, modern wild and ancient sheep of Turkey on the basis of their mtDNA in order to contribute to the understanding of the maternal evolutionary history of sheep by integrating the acquired data of the present study with the wealth of available data. Further genetic studies of modern and ancient samples of sheep from different regions and time periods of Anatolia and the Middle East will expand the understanding of both the early stages of the domestication process and the evolutionary history of domestic sheep.

## Supporting Information

Figure S1 **Mismatch distribution analyses of HPG E for three cases.** The cases (E1–3) were explained in the Materials and Methods section. Expected values were generated according to the sudden expansion model (Schneider and Excoffier, 1999).
(TIF)

Figure S2 **Spatial Pattern Analysis based on mtDNA haplogroups.** Spatial autocorrelation coefficients were represented by r values. Dashed lines represent the 95% CI. Vertical bars indicate 95% CI for defined distance class. Each vertical bar for a defined distance class outside the 95% CI indicates significant (p≤0.001) deviation, from the expectation of random distribution.
(TIF)

Table S1 **Locations, tail types, sample sizes, and haplogroup frequencies of the breeds.**
(DOC)

Table S2 **Coordinates of the sampled flocks, flock sample sizes, identities of the samples and haplogroup compositions of the samples.**
(DOC)

Table S3 **The mtDNA CR positions which were used to determine haplogroups of aDNA.**
(DOC)

Table S4 **Sequences employed to infer genetic relatedness of wild and domestic sheep on the basis of partial cytB sequences by median-joining network.**
(DOC)

Table S5 **Sequences used to construct neighbor joining tree and mismatch distributions and to compute genetic diversities.**
(DOC)

Table S6 **aDNA sample codes and their corresponding archaeological codes.**
(DOC)

Table S7 **The average number of nucleotide differences per site (Dxy) among the haplogroups of domestic sheep,** ***O. g. anatolica*** **and** ***O. g. musimon.***
(DOC)

Table S8 **Mean mismatch values, Fu's FS statistics and Tajima's D statistics of domestic haplogroups.**
(DOC)

Text S1 **Identification of ancient sheep samples.**
(DOC)

Text S2 **Study site for aDNA.**
(DOC)
