# Supplementary figures and images for: Mitochondrial DNA Diversity of Modern, Ancient and Wild Sheep (Ovis gmelinii anatolica) from Turkey: New Insights on the Evolutionary History of Sheep
Source: PLoS One. 2013 Dec 11;8(12):e81952. doi: 10.1371/journal.pone.0081952 (PMC3859546; doi:10.1371/journal.pone.0081952)

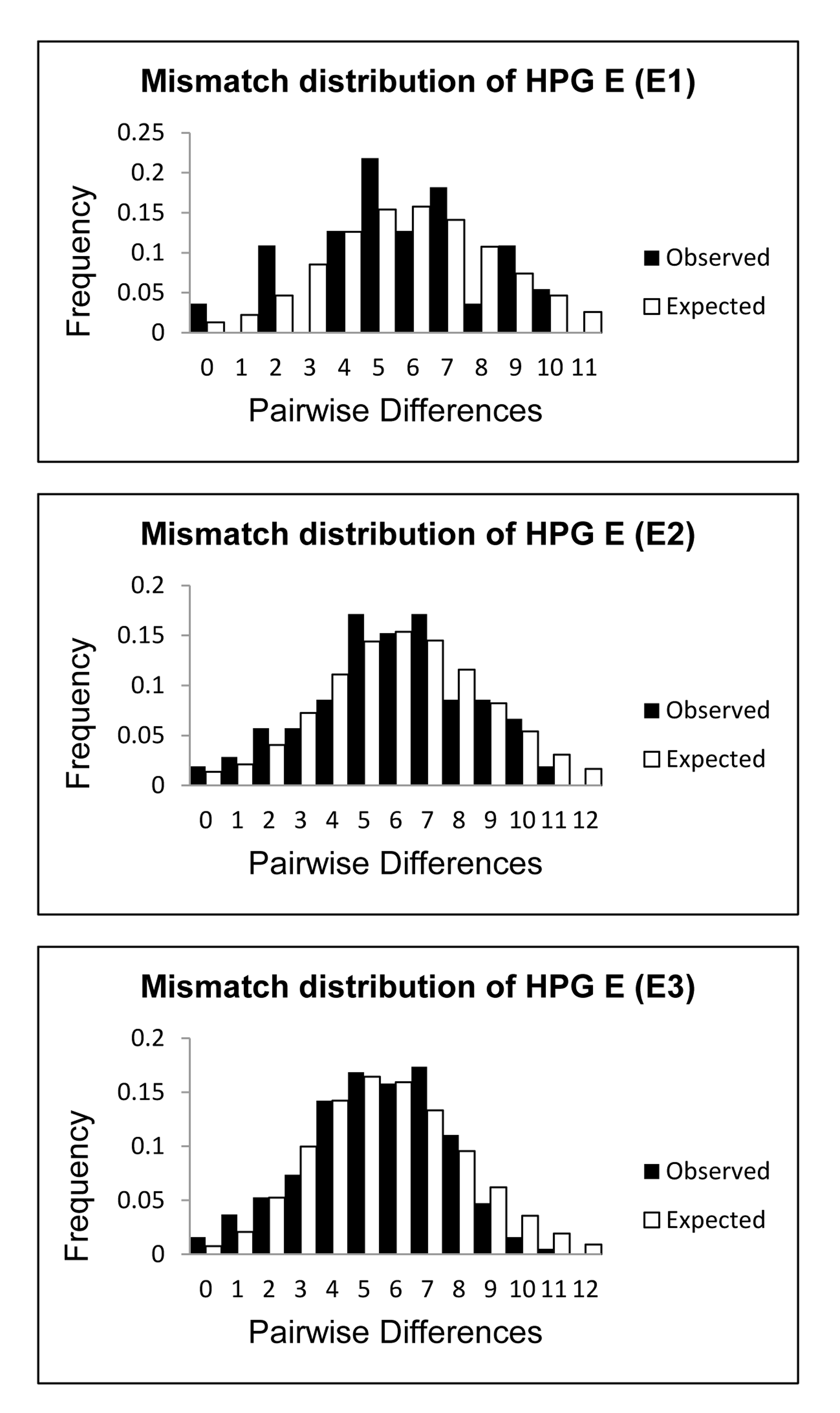

Supplement: Figure S1 — Mismatch distribution analyses of HPG E for three cases. The cases (E1–3) were explained in the Materials and Methods section. Expected values were generated according to the sudden expansion model (Schneider and Excoffier, 1999). (TIF) [file pone.0081952.s001.tif]

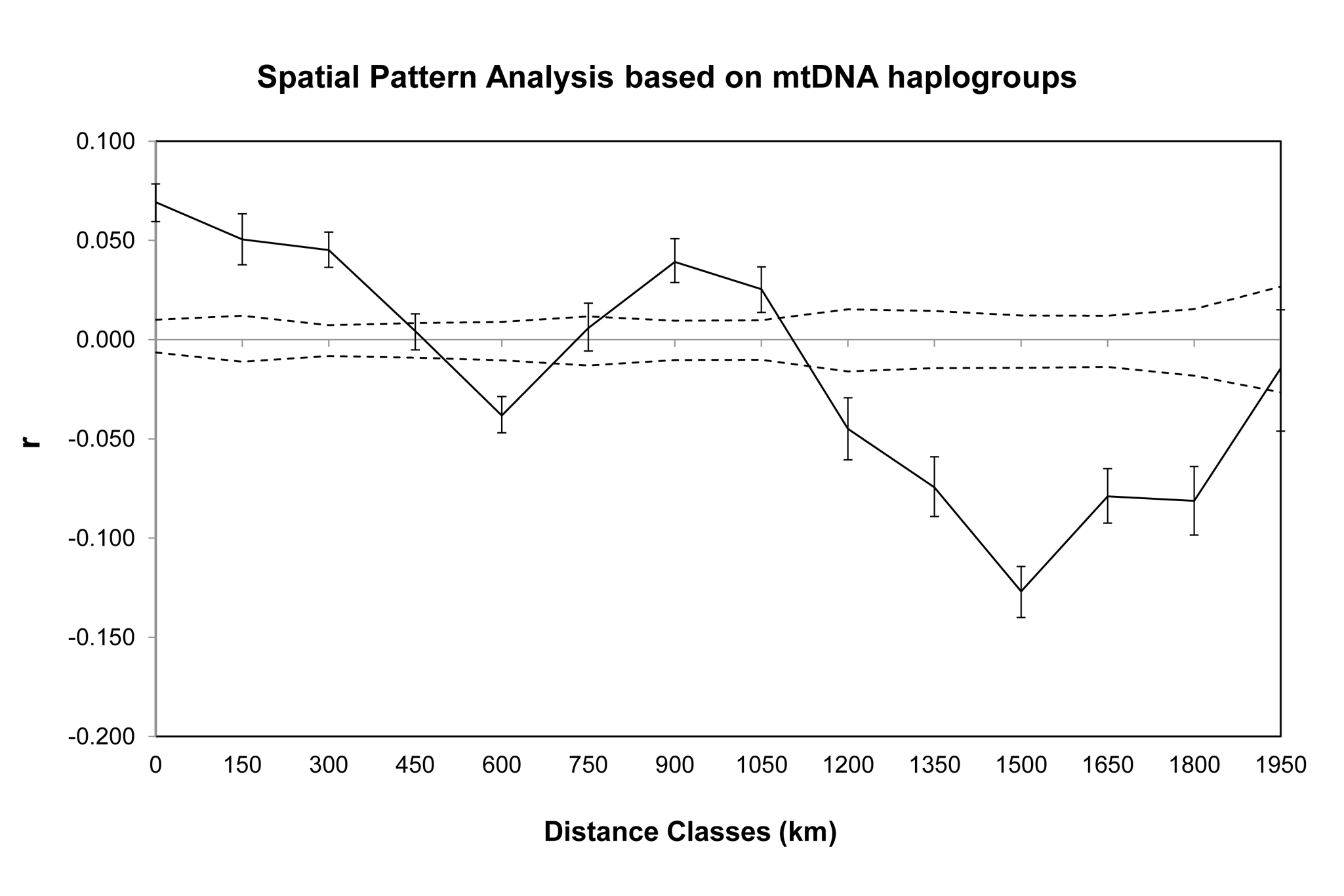

Supplement: Figure S2 — Spatial Pattern Analysis based on mtDNA haplogroups. Spatial autocorrelation coefficients were represented by r values. Dashed lines represent the 95% CI. Vertical bars indicate 95% CI for defined distance class. Each vertical bar for a defined distance class outside the 95% CI indicates significant (p≤0.001) deviation, from the expectation of random distribution. (TIF) [file pone.0081952.s002.tif]
