# Supplementary material for: Mitochondrial DNA Diversity of Modern, Ancient and Wild Sheep (Ovis gmelinii anatolica) from Turkey: New Insights on the Evolutionary History of Sheep
Source: PLoS One. 2013 Dec 11;8(12):e81952. doi: 10.1371/journal.pone.0081952 (PMC3859546; doi:10.1371/journal.pone.0081952)
Supplement: Table S1 — Locations, tail types, sample sizes, and haplogroup frequencies of the breeds. (DOC) [file pone.0081952.s003.doc]

**Table S1. Locations, tail types, sample sizes, and haplogroup frequencies of the breeds**

| **Breed name** | **Location** |  |  | **Tail type** | **Sample size** | **Haplogroup frequencies (%)** | | | | |
| --- | --- | --- | --- | --- | --- | --- | --- | --- | --- | --- |
|  | **Provinces** | **Latitude** | **Longitude** |  |  | **HPG A** | **HPG B** | **HPG C** | **HPG D** | **HPG E** |
| Karayaka | Tokat and Ordu | 40.4672 | 36.6369 | Thin | 50 | 18 | 74 | 6 |  | 2 |
| Akkaraman | Konya | 37.9166 | 32.8666 | Fat | 50 | 26 | 52 | 14 | 2 | 6 |
| Gökçeada | Çanakkale | 40.2044 | 25.9365 | Thin | 50 | 32 | 68 |  |  |  |
| Dağlıç | Afyon | 38.4144 | 30.4477 | Fat | 50 | 14 | 68 | 16 |  | 2 |
| Morkaraman | Erzurum | 39.8414 | 41.8270 | Fat | 50 | 40 | 30 | 22 |  | 8 |
| Kıvırcık | Kırklareli | 41.8698 | 27.5069 | Thin | 45 | 0 | 95.56 | 4.44 |  |  |
| İvesi | Şanlıurfa | 37.0700 | 39.0283 | Fat | 51 | 37.25 | 41.18 | 21.57 |  |  |
| Herik | Amasya | 40.8203 | 35.4966 | Semi fat | 49 | 12.25 | 81.63 | 6.12 |  |  |
| Karagül | Tokat | 40.3940 | 36.3491 | Fat | 50 | 26 | 64 | 6 |  | 4 |
| Hemşin | Artvin | 41.1972 | 42.0305 | Semi fat | 48 | 33.33 | 56.25 | 10.42 |  |  |
| Çineçaparı | Aydın | 37.6266 | 27.8333 | Fat | 40 | 10 | 77.5 | 12.5 |  |  |
| Sakız | İzmir | 38.2956 | 26.3508 | Semi fat | 49 | 4.08 | 87.76 | 8.16 |  |  |
| Norduz | Van | 38.0750 | 43.5133 | Fat | 46 | 28.26 | 45.65 | 23.91 | 2.18 |  |
| Total |  |  |  |  | 628 | 22 | 64.3 | 11.6 | 0.3 | 1.8 |
