# Supplementary material for: Mitochondrial DNA Diversity of Modern, Ancient and Wild Sheep (Ovis gmelinii anatolica) from Turkey: New Insights on the Evolutionary History of Sheep
Source: PLoS One. 2013 Dec 11;8(12):e81952. doi: 10.1371/journal.pone.0081952 (PMC3859546; doi:10.1371/journal.pone.0081952)
Supplement: Table S2 — Coordinates of the sampled flocks, flock sample sizes, identities of the samples and haplogroup compositions of the samples. (DOC) [file pone.0081952.s004.doc]

**Table S2. Coordinates of the sampled flocks, flock sample sizes, identities of the samples and haplogroup compositions of the samples**

| **Latitude** | **Longitude** | **Breed name** | **Sample size** | **Sample identities** | **Haplogroup composition** | | | | |
| --- | --- | --- | --- | --- | --- | --- | --- | --- | --- |
|  |  |  |  |  | **A** | **B** | **C** | **D** | **E** |
| 40.6391 | 36.8200 | Karayaka | 7 | KRY1,3-8 | 1 | 3 | 2 |  | 1 |
| 40.5426 | 36.6566 | Karayaka | 3 | KRY2,21,27 | 1 | 2 |  |  |  |
| 40.5738 | 37.2129 | Karayaka | 6 | KRY9-12,18,19 | 3 | 3 |  |  |  |
| 40.5417 | 37.2936 | Karayaka | 4 | KRY13,48,49,50 |  | 4 |  |  |  |
| 40.5410 | 36.6067 | Karayaka | 2 | KRY14,25 | 1 | 1 |  |  |  |
| 40.1455 | 35.9774 | Karayaka | 1 | KRY15 |  | 1 |  |  |  |
| 40.1670 | 35.7256 | Karayaka | 2 | KRY16,34 |  | 2 |  |  |  |
| 40.3219 | 36.4705 | Karayaka | 3 | KRY17,20,28 | 1 | 2 |  |  |  |
| 40.5359 | 36.7333 | Karayaka | 2 | KRY22,40 |  | 2 |  |  |  |
| 40.1685 | 36.2490 | Karayaka | 3 | KRY23,24,41 | 1 | 2 |  |  |  |
| 40.5393 | 36.5748 | Karayaka | 1 | KRY26 |  | 1 |  |  |  |
| 40.7404 | 36.5955 | Karayaka | 9 | KRY29-33, 35-38 | 1 | 8 |  |  |  |
| 40.3351 | 36.5523 | Karayaka | 1 | KRY39 |  | 1 |  |  |  |
| 40.7503 | 37.4493 | Karayaka | 6 | KRY42-47 |  | 5 | 1 |  |  |
| 37.9667 | 32.9000 | Akkaraman | 14 | AKK1-6, 22 ,26-31 ,47 | 6 | 8 |  |  |  |
| 37.9000 | 32.8833 | Akkaraman | 23 | AKK7-18, 32-42 | 3 | 11 | 6 | 1 | 2 |
| 37.8333 | 32.7833 | Akkaraman | 13 | AKK19-21,23-25,43-46,48-50 | 4 | 7 | 1 |  | 1 |
| 40.2321 | 25.9446 | Gökçeada | 1 | GOK1 | 1 |  |  |  |  |
| 40.2152 | 25.9128 | Gökçeada | 12 | GOK2-7,31-35 | 3 | 9 |  |  |  |
| 40.1297 | 25.9493 | Gökçeada | 17 | GOK8-9, 36-50 | 2 | 15 |  |  |  |
| 40.2148 | 25.9373 | Gökçeada | 16 | GOK10-25 | 9 | 7 |  |  |  |
| 40.2305 | 25.9388 | Gökçeada | 5 | GOK26-30 | 1 | 4 |  |  |  |
| 38.4700 | 31.0500 | Dağlıç | 7 | DAG1-3,24,34,58-59 | 1 | 5 | 1 |  |  |
| 38.4600 | 31.0600 | Dağlıç | 2 | DAG4-5 |  | 2 |  |  |  |
| 38.4200 | 31.0500 | Dağlıç | 3 | DAG6-8 |  | 3 |  |  |  |
| 38.2700 | 30.2600 | Dağlıç | 4 | DAG9,25,35,57 | 3 |  | 1 |  |  |
| 38.0800 | 30.1500 | Dağlıç | 8 | DAG10-13, 20, 47-49 |  | 6 | 2 |  |  |
| 38.5600 | 30.3000 | Dağlıç | 3 | DAG14, 55-56 | 1 | 1 | 1 |  |  |
| 38.5900 | 30.3000 | Dağlıç | 5 | DAG15-18,50 | 1 | 4 |  |  |  |
| 38.5100 | 30.3100 | Dağlıç | 1 | DAG19 |  | 1 |  |  |  |
| 38.3700 | 29.5500 | Dağlıç | 17 | DAG21-23,31-33,36-46 | 1 | 12 | 3 |  | 1 |
| 39.7900 | 41.0900 | Morkaraman | 1 | MRK1 | 1 |  |  |  |  |
| 39.9400 | 41.6000 | Morkaraman | 1 | MRK3 |  | 1 |  |  |  |
| 39.8900 | 41.6700 | Morkaraman | 2 | MRK14,18 |  |  | 1 |  | 1 |
| 40.0400 | 41.0900 | Morkaraman | 2 | MRK17,21 | 1 | 1 |  |  |  |
| 39.9400 | 41.7200 | Morkaraman | 1 | MRK23 | 1 |  |  |  |  |
| 39.9200 | 41.5300 | Morkaraman | 2 | MRK5,24 |  | 1 |  |  | 1 |
| 39.8900 | 41.7300 | Morkaraman | 1 | MRK25 |  | 1 |  |  |  |
| 39.9900 | 41.4900 | Morkaraman | 3 | MRK9-10,27 | 1 | 1 | 1 |  |  |
| 39.9284 | 41.4982 | Morkaraman | 3 | MRK8,33,35 | 1 | 1 | 1 |  |  |
| 39.7700 | 41.7600 | Morkaraman | 3 | MRK2,6,39 | 3 |  |  |  |  |
| 40.0145 | 41.8221 | Morkaraman | 5 | MRK7,30-31,41-42 | 2 |  | 3 |  |  |
| 40.0700 | 41.8000 | Morkaraman | 5 | MRK4,15,26,38,43 | 3 | 1 | 1 |  |  |
| 39.7000 | 42.3900 | Morkaraman | 6 | MRK11-13,40,45,47 | 1 | 4 | 1 |  |  |
| 40.0300 | 41.6800 | Morkaraman | 12 | MRK16,19-20,22,29,32,34,36-37,44,48-49 | 5 | 3 | 1 |  | 3 |
| 39.8642 | 41.9165 | Morkaraman | 3 | MRK28,46,50 | 1 |  | 2 |  |  |
| 41.8377 | 27.4681 | Kıvırcık | 38 | KIV1-3,6-12,14-15,20-51 |  | 36 | 2 |  |  |
| 41.9020 | 27.5458 | Kıvırcık | 7 | KIV4-5,13,16-19 |  | 7 |  |  |  |
| 36.9900 | 39.1400 | İvesi | 6 | IVE1,13,25-27,41 | 2 | 2 | 2 |  |  |
| 37.0600 | 39.2600 | İvesi | 20 | IVE2-10,16,30,43-50,52 | 4 | 12 | 4 |  |  |
| 37.1400 | 38.7900 | İvesi | 16 | IVE11,19-20,22-24,31-39,51 | 10 | 3 | 3 |  |  |
| 37.1200 | 38.9000 | İvesi | 1 | IVE12 |  | 1 |  |  |  |
| 37.0100 | 39.1500 | İvesi | 3 | IVE14-15,28 | 1 |  | 2 |  |  |
| 37.1000 | 38.9300 | İvesi | 4 | IVE17-18,21,29,42 | 2 | 3 |  |  |  |
| 40.8908 | 35.5547 | Herik | 1 | HER2 |  | 1 |  |  |  |
| 40.8428 | 35.6131 | Herik | 6 | HER3,15-17,22-23 | 1 | 5 |  |  |  |
| 40.7908 | 35.4658 | Herik | 5 | HER6,9,12,21,24 | 1 | 4 |  |  |  |
| 40.8406 | 35.1761 | Herik | 1 | HER7 |  | 1 |  |  |  |
| 40.8686 | 35.4719 | Herik | 5 | HER8,14,18-20 |  | 5 |  |  |  |
| 40.7047 | 35.5156 | Herik | 1 | HER10 |  | 1 |  |  |  |
| 40.8619 | 35.4692 | Herik | 5 | HER1,4-5,11,13 | 1 | 3 | 1 |  |  |
| 40.8486 | 35.4812 | Herik | 2 | HER25-26 |  | 1 | 1 |  |  |
| 40.8896 | 35.5550 | Herik | 2 | HER30-31 | 1 | 1 |  |  |  |
| 40.9016 | 35.5201 | Herik | 2 | HER32-33 |  | 2 |  |  |  |
| 40.8749 | 35.4597 | Herik | 4 | HER27-29,34 |  | 4 |  |  |  |
| 40.7562 | 35.5596 | Herik | 1 | HER35 |  | 1 |  |  |  |
| 40.7532 | 35.6702 | Herik | 4 | HER36-39 |  | 3 | 1 |  |  |
| 40.7297 | 35.7045 | Herik | 1 | HER40 |  | 1 |  |  |  |
| 40.7323 | 35.2910 | Herik | 1 | HER41 |  | 1 |  |  |  |
| 40.8682 | 35.4691 | Herik | 5 | HER42-46 | 1 | 4 |  |  |  |
| 40.7914 | 35.4660 | Herik | 3 | HER47-49 | 1 | 2 |  |  |  |
| 40.6000 | 36.1500 | Karagül | 2 | KRG1-2 | 1 |  | 1 |  |  |
| 40.4000 | 36.0300 | Karagül | 2 | KRG3-4 |  | 2 |  |  |  |
| 40.2900 | 36.3900 | Karagül | 22 | KRG5-13,17-25,33,49-51 | 5 | 13 | 2 |  | 2 |
| 40.3000 | 36.4300 | Karagül | 11 | KRG14-16,45-48,52-55 | 5 | 6 |  |  |  |
| 40.4600 | 36.7500 | Karagül | 2 | KRG31-32 | 1 | 1 |  |  |  |
| 40.3350 | 36.2167 | Karagül | 5 | KRG34-38 | 1 | 4 |  |  |  |
| 40.4639 | 36.3936 | Karagül | 6 | KRG39-44 |  | 6 |  |  |  |
| 41.1103 | 42.0721 | Hemşin | 9 | HEM1,3-4,10,12,25,38,41,43 | 5 | 4 |  |  |  |
| 41.0406 | 42.2116 | Hemşin | 8 | HEM2,9,17,21,24,27,36,40 | 2 | 5 | 1 |  |  |
| 41.0741 | 41.9804 | Hemşin | 4 | HEM5,18,32,48 |  | 3 | 1 |  |  |
| 41.1431 | 42.0617 | Hemşin | 2 | HEM6,42 | 1 | 1 |  |  |  |
| 41.1189 | 42.1512 | Hemşin | 2 | HEM7,37 |  | 2 |  |  |  |
| 41.4407 | 41.6168 | Hemşin | 3 | HEM8,45-46 |  | 3 |  |  |  |
| 41.0374 | 42.1512 | Hemşin | 1 | HEM11 |  | 1 |  |  |  |
| 41.1135 | 42.1531 | Hemşin | 1 | HEM13 | 1 |  |  |  |  |
| 41.0444 | 42.1603 | Hemşin | 2 | HEM14,34 | 2 |  |  |  |  |
| 41.0392 | 42.2135 | Hemşin | 3 | HEM15,29,31 | 1 | 2 |  |  |  |
| 41.1605 | 42.0652 | Hemşin | 6 | HEM16,19,22,33,35,44 | 2 | 2 | 2 |  |  |
| 41.2994 | 42.3169 | Hemşin | 4 | HEM20,23,26,30 | 1 | 3 |  |  |  |
| 41.0445 | 42.0944 | Hemşin | 1 | HEM28 |  | 1 |  |  |  |
| 41.0367 | 42.1513 | Hemşin | 1 | HEM39 | 1 |  |  |  |  |
| 41.1114 | 42.0827 | Hemşin | 1 | HEM47 |  |  | 1 |  |  |
| 37.7500 | 27.6900 | Çineçaparı | 22 | CIC1-5,11,25-41 | 2 | 18 | 2 |  |  |
| 37.5900 | 27.7200 | Çineçaparı | 5 | CIC6-10 | 2 | 3 |  |  |  |
| 37.5400 | 28.0900 | Çineçaparı | 13 | CIC12-24 |  | 10 | 3 |  |  |
| 38.3408 | 26.2908 | Sakız | 10 | SAK1-5,26-30 |  | 10 |  |  |  |
| 38.2914 | 26.3331 | Sakız | 9 | SAK6-10,32-35 |  | 9 |  |  |  |
| 38.3000 | 26.4500 | Sakız | 17 | SAK11-16,36-46 | 1 | 16 |  |  |  |
| 38.3000 | 26.4167 | Sakız | 3 | SAK17-18,47 |  |  | 3 |  |  |
| 38.2500 | 26.3000 | Sakız | 3 | SAK19,49-50 |  | 3 |  |  |  |
| 38.2833 | 26.2833 | Sakız | 6 | SAK20-25 | 1 | 5 |  |  |  |
| 38.3167 | 26.4000 | Sakız | 1 | SAK48 |  |  | 1 |  |  |
| 38.0300 | 43.3700 | Norduz | 6 | NOR1-2,12-13,18,43 | 2 | 2 | 2 |  |  |
| 37.8700 | 43.4400 | Norduz | 13 | NOR3-11,14-17 | 3 | 7 | 3 |  |  |
| 38.0400 | 44.0100 | Norduz | 8 | NOR19-25,42 | 2 | 4 | 2 |  |  |
| 38.3200 | 43.4100 | Norduz | 19 | NOR26-41,44-46 | 6 | 8 | 4 | 1 |  |
