# Supplementary material for: Mitochondrial DNA Diversity of Modern, Ancient and Wild Sheep (Ovis gmelinii anatolica) from Turkey: New Insights on the Evolutionary History of Sheep
Source: PLoS One. 2013 Dec 11;8(12):e81952. doi: 10.1371/journal.pone.0081952 (PMC3859546; doi:10.1371/journal.pone.0081952)
Supplement: Table S3 — The mtDNA CR positions which were used to determine haplogroups of aDNA. (DOC) [file pone.0081952.s005.doc]

**Table S3. The mtDNA CR positions which were used to determine haplogroups of aDNA**

| **HPG** | **Reference Sequence** | **HPG discriminating**  **positions on AF010406** | | | | |
| --- | --- | --- | --- | --- | --- | --- |
|  |  | 15459 | 15476 | 15484 | 15509 | 15512 |
| HPG A | HM236174 | T | T | A | A | T |
| HPG B | HM236176 | C | T | G | A | T |
| HPG C | HM236178 | C | T | G | G | T |
| HPG D | HM236180 | C | T | G | A | C |
| HPG E | HM236182 | C | C | G | G | T |
