# Supplementary material for: Mitochondrial DNA Diversity of Modern, Ancient and Wild Sheep (Ovis gmelinii anatolica) from Turkey: New Insights on the Evolutionary History of Sheep
Source: PLoS One. 2013 Dec 11;8(12):e81952. doi: 10.1371/journal.pone.0081952 (PMC3859546; doi:10.1371/journal.pone.0081952)
Supplement: Table S4 — Sequences employed to infer genetic relatedness of wild and domestic sheep on the basis of partial cytB sequences by median-joining network. (DOC) [file pone.0081952.s006.doc]

**Table S4. Sequences employed to infer genetic relatedness of wild and domestic sheep on the basis of partial *cytB* sequences by median-joining network**

| **Accession** | **Sample Name** | **Sample Name (according to Rezaei et al. (2010))** | **Haplotype** | **Species name** | **Country** | **References** |
| --- | --- | --- | --- | --- | --- | --- |
| **HM236174** | cl122 |  | H_1 | *Ovis aries, HPG A* | Australia | Meadows et al., 2011 |
| **HM236175** | r359 |  | H_1 | *Ovis aries, HPG A* | Australia | Meadows et al., 2011 |
| **HM236176** | kk1 |  | H_2 | *Ovis aries, HPG B* | Turkey | Meadows et al., 2011 |
| **HM236177** | kk2 |  | H_2 | *Ovis aries, HPG B* | Turkey | Meadows et al., 2011 |
| **HM236178** | kk12 |  | H_3 | *Ovis aries, HPG C* | Turkey | Meadows et al., 2011 |
| **HM236179** | mk4 |  | H_3 | *Ovis aries, HPG C* | Turkey | Meadows et al., 2011 |
| **HM236180** | mk3 |  | H_4 | *Ovis aries, HPG D* | Turkey | Meadows et al., 2011 |
| **HM236181** | mk9 |  | H_4 | *Ovis aries, HPG D* | Turkey | Meadows et al., 2011 |
| **HM236182** | aw25 |  | H_5 | *Ovis aries, HPG E* | Israel | Meadows et al., 2011 |
| **HM236183** | tj6 |  | H_6 | *Ovis aries, HPG E* | Turkey | Meadows et al., 2011 |
| **KF677296** | DAG21 |  | H_7 | *Ovis aries, HPG A* | Turkey | Present Study |
| **KF677299** | HER24 |  | H_1 | *Ovis aries, HPG A* | Turkey | Present Study |
| **KF677302** | HEM17 |  | H_2 | *Ovis aries, HPG B* | Turkey | Present Study |
| **KF677295** | GOK8 |  | H_2 | *Ovis aries, HPG B* | Turkey | Present Study |
| **KF677298** | IVE14 |  | H_8 | *Ovis aries, HPG C* | Turkey | Present Study |
| **KF677300** | NOR18 |  | H_8 | *Ovis aries, HPG C* | Turkey | Present Study |
| **KF677294** | AKK39 |  | H_4 | *Ovis aries, HPG D* | Turkey | Present Study |
| **KF677301** | NOR45 |  | H_9 | *Ovis aries, HPG D* | Turkey | Present Study |
| **KF677297** | DAG36 |  | H_5 | *Ovis aries, HPG E* | Turkey | Present Study |
| **KF677303** | MRK24 |  | H_5 | *Ovis aries, HPG E* | Turkey | Present Study |
| **HM236184** | h1 |  | H_2 | *Ovis gmelinii musimon* | Germany | Meadows et al., 2011 |
| **HM236185** | h2 |  | H_2 | *Ovis gmelinii musimon* | Germany | Meadows et al., 2011 |
| **EU365977** | OomFr1 | OM1 | H_10 | *Ovis gmelinii musimon* | France | Rezaei et al., 2010 |
| **EU365990** | OomFr2 | OM2 | H_11 | *Ovis gmelinii musimon* | France | Rezaei et al., 2010 |
| **FR873152** | H4 |  | H_2 | *Ovis gmelinii musimon* | Italy | Barbanera et al., 2012 |
| **FR873150** | H2 |  | H_12 | *Ovis gmelinii musimon* | Italy | Barbanera et al., 2012 |
| **FR873151** | H3 |  | H_10 | *Ovis gmelinii musimon* | Italy | Barbanera et al., 2012 |
| **FR873149** | H1 |  | H_13 | *Ovis gmelinii ophion* | Cyprus | Barbanera et al., 2012 |
| **EU365973** | OoaTk1 | OOA1 | H_1 | *Ovis gmelinii anatolica* | Turkey | Rezaei et al., 2010 |
| **EU365987** | OoaTk4 | OOA2 | H_14 | *Ovis gmelinii anatolica* | Turkey | Rezaei et al., 2010 |
| **FJ936185** | OOA3 |  | H_15 | *Ovis gmelinii anatolica* | Turkey | Rezaei et al., 2010 |
| **KF677305** | OGA14 |  | H_15 | *Ovis gmelinii anatolica* | Turkey | Present Study |
| **KF677307** | OGA21 |  | H_15 | *Ovis gmelinii anatolica* | Turkey | Present Study |
| **KF677304** | OGA9 |  | H_1 | *Ovis gmelinii anatolica* | Turkey | Present Study |
| **KF677306** | OGA18 |  | H_1 | *Ovis gmelinii anatolica* | Turkey | Present Study |
| **EU366040** | OogAr1 | OOG1 | H_16 | *Ovis gmelinii gmelini* | Armenia | Rezaei et al., 2010 |
| **FJ936190** | OOG2 |  | H_5 | *Ovis gmelinii gmelini* | Iran | Rezaei et al., 2010 |
| **FJ936200** | OOG4 |  | H_17 | *Ovis gmelinii gmelini* | Iran | Rezaei et al., 2010 |
| **FJ936201** | OOG5 |  | H_18 | *Ovis gmelinii gmelini* | Iran | Rezaei et al., 2010 |
| **EU365979** | OogSn1 | OOG6 | H_13 | *Ovis gmelinii gmelini* | Iran | Rezaei et al., 2010 |
| **EU365980** | OogSn2 | OOG7 | H_19 | *Ovis gmelinii gmelini* | Iran | Rezaei et al., 2010 |
| **FJ936202** | OOG8 |  | H_5 | *Ovis gmelinii gmelini* | Iran | Rezaei et al., 2010 |
| **EU365996** | OogBi1 | OOG9 | H_20 | *Ovis gmelinii gmelini* | Iran | Rezaei et al., 2010 |
| **EU365997** | OogBi2 | OOG10 | H_21 | *Ovis gmelinii gmelini* | Iran | Rezaei et al., 2010 |
| **FJ936203** | OOG9 | OOG11 | H_20 | *Ovis gmelinii gmelini* | Iran | Rezaei et al., 2010 |
| **FJ936186** | OOG11 | OOG12 | H_13 | *Ovis gmelinii gmelini* | Iran | Rezaei et al., 2010 |
| **FJ936187** | OOG12 | OOG13 | H_21 | *Ovis gmelinii gmelini* | Iran | Rezaei et al., 2010 |
| **FJ936188** | OOG13 |  | H_1 | *Ovis gmelinii gmelini* | Iran | Rezaei et al., 2010 |
| **EU366015** | OogMa2 | OOG14 | H_22 | *Ovis gmelinii gmelini* | Iran | Rezaei et al., 2010 |
| **FJ936189** | OOG15 |  | H_13 | *Ovis gmelinii gmelini* | Iran | Rezaei et al., 2010 |
| **EU365975** | OogMk1 | OOG16 | H_2 | *Ovis gmelinii gmelini* | Iran | Rezaei et al., 2010 |
| **EU365989** | OogMk3 | OOG17 | H_23 | *Ovis gmelinii gmelini* | Iran | Rezaei et al., 2010 |
| **EU365998** | OogMk4 | OOG18 | H_24 | *Ovis gmelinii gmelini* | Iran | Rezaei et al., 2010 |
| **EU365999** | OogMk5 | OOG19 | H_5 | *Ovis gmelinii gmelini* | Iran | Rezaei et al., 2010 |
| **EU366000** | OogMk6 | OOG20 | H_24 | *Ovis gmelinii gmelini* | Iran | Rezaei et al., 2010 |
| **FJ936191** | OOG22 |  | H_5 | *Ovis gmelinii gmelini* | Iran | Rezaei et al., 2010 |
| **FJ936192** | OOG23 |  | H_5 | *Ovis gmelinii gmelini* | Iran | Rezaei et al., 2010 |
| **EU366002** | OogZa1 | OOG24 | H_25 | *Ovis gmelinii gmelini* | Iran | Rezaei et al., 2010 |
| **FJ936193** | OOG25 |  | H_13 | *Ovis gmelinii gmelini* | Iran | Rezaei et al., 2010 |
| **FJ936194** | OOG26 |  | H_1 | *Ovis gmelinii gmelini* | Iran | Rezaei et al., 2010 |
| **FJ936195** | OOG27 |  | H_21 | *Ovis gmelinii gmelini* | Iran | Rezaei et al., 2010 |
| **EU366003** | OogGa1 | OOG28 | H_26 | *Ovis gmelinii gmelini* | Iran | Rezaei et al., 2010 |
| **EU366053** | OogGa2 | OOG29 | H_27 | *Ovis gmelinii gmelini* | Iran | Rezaei et al., 2010 |
| **FJ936197** | OOG31 |  | H_13 | *Ovis gmelinii gmelini* | Iran | Rezaei et al., 2010 |
| **FJ936198** | OOG32 |  | H_1 | *Ovis gmelinii gmelini* | Turkey | Rezaei et al., 2010 |
| **FJ936199** | OOG33 |  | H_1 | *Ovis gmelinii gmelini* | Turkey | Rezaei et al., 2010 |
| **EU366016** | OgiAz1 | OOI1 | H_28 | *Ovis gmelinii isphahanica* | Iran | Rezaei et al., 2010 |
| **EU365976** | OoiAz2 | OOI2 | H_1 | *Ovis gmelinii isphahanica* | Iran | Rezaei et al., 2010 |
| **FJ936204** | OOI3 |  | H_1 | *Ovis gmelinii isphahanica* | Iran | Rezaei et al., 2010 |
| **FJ936205** | OOI5 |  | H_29 | *Ovis gmelinii isphahanica* | Iran | Rezaei et al., 2010 |
| **FJ936206** | OOI6 |  | H_29 | *Ovis gmelinii isphahanica* | Iran | Rezaei et al., 2010 |
| **FJ936209** | OOL1 |  | H_13 | *Ovis gmelinii laristanica* | Iran | Rezaei et al., 2010 |
| **FJ936210** | OOL2 |  | H_5 | *Ovis gmelinii laristanica* | Iran | Rezaei et al., 2010 |
| **FJ936211** | OOS1 |  | H_30 | *Ovis gmelinii population Sh* | Iran | Rezaei et al., 2010 |
| **FJ936207** | OOKo1 |  | H_31 | *Ovis gmelinii population Ko* | Iran | Rezaei et al., 2010 |
| **FJ936208** | OOKo2 |  | H_8 | *Ovis gmelinii population Ko* | Iran | Rezaei et al., 2010 |
| **AJ867261** | J20 |  | H_32 | *Ovis gmelinii anatolica* | Turkey | Bunch et al., 2006 |
| **EU365991** | OogKh1 | OxV1 | H_33 | *Ovis gmelinii x vignei* | Iran | Rezaei et al., 2010 |
| **EU366009** | OogKh2 | OxV2 | H_34 | *Ovis gmelinii x vignei* | Iran | Rezaei et al., 2010 |
| **EU366068** | OogKh3 | OxV3 | H_35 | *Ovis gmelinii x vignei* | Iran | Rezaei et al., 2010 |
| **EU365978** | OolBa1 | OxV4 | H_36 | *Ovis gmelinii x vignei* | Iran | Rezaei et al., 2010 |
| **EU365982** | OolBa3 | OxV5 | H_37 | *Ovis gmelinii x vignei* | Iran | Rezaei et al., 2010 |
| **FJ936233** | OxV6 |  | H_36 | *Ovis gmelinii x vignei* | Iran | Rezaei et al., 2010 |
| **FJ936234** | OxV7 |  | H_38 | *Ovis gmelinii x vignei* | Iran | Rezaei et al., 2010 |
| **FJ936235** | OxV8 |  | H_38 | *Ovis gmelinii x vignei* | Iran | Rezaei et al., 2010 |
| **EU366019** | OvaPa1 | OxV9 | H_39 | *Ovis gmelinii x vignei* | Iran | Rezaei et al., 2010 |
| **EU366020** | OvaPa2 | OxV10 | H_39 | *Ovis gmelinii x vignei* | Iran | Rezaei et al., 2010 |
| **EU366021** | OvaPa3 | OxV11 | H_40 | *Ovis gmelinii x vignei* | Iran | Rezaei et al., 2010 |
| **FJ936222** | OxV12 |  | H_41 | *Ovis gmelinii x vignei* | Iran | Rezaei et al., 2010 |
| **FJ936223** | OxV13 |  | H_34 | *Ovis gmelinii x vignei* | Iran | Rezaei et al., 2010 |
| **FJ936224** | OxV14 |  | H_39 | *Ovis gmelinii x vignei* | Iran | Rezaei et al., 2010 |
| **EU366005** | OvaTu1 | OxV15 | H_41 | *Ovis gmelinii x vignei* | Iran | Rezaei et al., 2010 |
| **EU366006** | OvaTu2 | OxV16 | H_42 | *Ovis gmelinii x vignei* | Iran | Rezaei et al., 2010 |
| **EU366007** | OvaTu3 | OxV17 | H_43 | *Ovis gmelinii x vignei* | Iran | Rezaei et al., 2010 |
| **EU366026** | OvkKe1 | OxV18 | H_44 | *Ovis gmelinii x vignei* | Iran | Rezaei et al., 2010 |
| **EU366027** | OvkKe2 | OxV19 | H_45 | *Ovis gmelinii x vignei* | Iran | Rezaei et al., 2010 |
| **EU366028** | OvkKe3 | OxV20 | H_46 | *Ovis gmelinii x vignei* | Iran | Rezaei et al., 2010 |
| **EU366029** | OvkKe4 | OxV21 | H_47 | *Ovis gmelinii x vignei* | Iran | Rezaei et al., 2010 |
| **EU366030** | OvkKe5 | OxV22 | H_47 | *Ovis gmelinii x vignei* | Iran | Rezaei et al., 2010 |
| **EU366035** | OvkKe6 | OxV23 | H_48 | *Ovis gmelinii x vignei* | Iran | Rezaei et al., 2010 |
| **FJ936225** | OxV24 |  | H_49 | *Ovis gmelinii x vignei* | Iran | Rezaei et al., 2010 |
| **FJ936226** | OxV25 |  | H_45 | *Ovis gmelinii x vignei* | Iran | Rezaei et al., 2010 |
| **EU366031** | OvkYa1 | OxV26 | H_50 | *Ovis gmelinii x vignei* | Iran | Rezaei et al., 2010 |
| **EU366032** | OvkYa2 | OxV27 | H_51 | *Ovis gmelinii x vignei* | Iran | Rezaei et al., 2010 |
| **EU366022** | OvaKa1 | OxV28 | H_49 | *Ovis gmelinii x vignei* | Iran | Rezaei et al., 2010 |
| **EU366023** | OvaKa2 | OxV29 | H_52 | *Ovis gmelinii x vignei* | Iran | Rezaei et al., 2010 |
| **EU366024** | OvaKa3 | OxV30 | H_49 | *Ovis gmelinii x vignei* | Iran | Rezaei et al., 2010 |
| **EU366025** | OvaKa4 | OxV31 | H_53 | *Ovis gmelinii x vignei* | Iran | Rezaei et al., 2010 |
| **FJ936227** | OxV32 |  | H_49 | *Ovis gmelinii x vignei* | Iran | Rezaei et al., 2010 |
| **FJ936228** | OxV33 |  | H_34 | *Ovis gmelinii x vignei* | Iran | Rezaei et al., 2010 |
| **FJ936229** | OxV34 |  | H_39 | *Ovis gmelinii x vignei* | Iran | Rezaei et al., 2010 |
| **FJ936230** | OxV35 |  | H_41 | *Ovis gmelinii x vignei* | Iran | Rezaei et al., 2010 |
| **FJ936231** | OxV36 |  | H_43 | *Ovis gmelinii x vignei* | Iran | Rezaei et al., 2010 |
| **FJ936232** | OxV37 |  | H_43 | *Ovis gmelinii x vignei* | Iran | Rezaei et al., 2010 |
| **FJ936212** | OVA12 |  | H_54 | *Ovis vignei arkal* | Iran | Rezaei et al., 2010 |
| **FJ936220** | OVV3 |  | H_55 | *Ovis vignei vignei* | Pakistan | Rezaei et al., 2010 |
| **AF242349** |  |  | H_56 | *Ovis ammon ammon* |  | Hiendleder and Kaupe, Unpublished |
| **DQ097407** | TUJ14 |  | H_2 | *Ovis aries, HPG B* | Turkey | Pedrosa et al., 2005 |
| **DQ097408** | KAR13 |  | H_57 | *Ovis aries, HPG B* | Turkey | Pedrosa et al., 2005 |
| **DQ097409** | HEM13 |  | H_58 | *Ovis aries, HPG B* | Turkey | Pedrosa et al., 2005 |
| **DQ097410** | HEM06 |  | H_59 | *Ovis aries, HPG B* | Turkey | Pedrosa et al., 2005 |
| **DQ097411** | TUJ11 |  | H_60 | *Ovis aries, HPG B* | Turkey | Pedrosa et al., 2005 |
| **DQ097412** | AKA02 |  | H_61 | *Ovis aries, HPG B* | Turkey | Pedrosa et al., 2005 |
| **DQ097413** | TUJ12 |  | H_2 | *Ovis aries, HPG B* | Turkey | Pedrosa et al., 2005 |
| **DQ097414** | TUJ08 |  | H_62 | *Ovis aries, HPG B* | Turkey | Pedrosa et al., 2005 |
| **DQ097415** | AKA01 |  | H_1 | *Ovis aries, HPG A* | Turkey | Pedrosa et al., 2005 |
| **DQ097416** | KAR09 |  | H_63 | *Ovis aries, HPG A* | Turkey | Pedrosa et al., 2005 |
| **DQ097417** | KAR04 |  | H_64 | *Ovis aries, HPG A* | Turkey | Pedrosa et al., 2005 |
| **DQ097418** | MOR04 |  | H_65 | *Ovis aries, HPG A* | Turkey | Pedrosa et al., 2005 |
| **DQ097419** | MOR02 |  | H_66 | *Ovis aries, HPG A* | Turkey | Pedrosa et al., 2005 |
| **DQ097420** | MOR09 |  | H_67 | *Ovis aries, HPG A* | Turkey | Pedrosa et al., 2005 |
| **DQ097421** | MOR11 |  | H_68 | *Ovis aries, HPG A* | Turkey | Pedrosa et al., 2005 |
| **DQ097422** | MOR13 |  | H_69 | *Ovis aries, HPG A* | Turkey | Pedrosa et al., 2005 |
| **DQ097423** | AKA06 |  | H_3 | *Ovis aries, HPG C* | Turkey | Pedrosa et al., 2005 |
| **DQ097424** | HEM01 |  | H_70 | *Ovis aries, HPG C* | Turkey | Pedrosa et al., 2005 |
| **DQ097425** | KAR02 |  | H_71 | *Ovis aries, HPG C* | Turkey | Pedrosa et al., 2005 |
| **DQ097426** | AKA07 |  | H_72 | *Ovis aries, HPG C* | Turkey | Pedrosa et al., 2005 |
| **DQ097427** | AKA10 |  | H_8 | *Ovis aries, HPG C* | Turkey | Pedrosa et al., 2005 |
| **DQ097429** | MOR12 |  | H_73 | *Ovis aries, HPG C* | Turkey | Pedrosa et al., 2005 |
| **DQ097430** | KAR15 |  | H_5 | *Ovis aries, HPG E* | Turkey | Pedrosa et al., 2005 |
