# Supplementary material for: Mitochondrial DNA Diversity of Modern, Ancient and Wild Sheep (Ovis gmelinii anatolica) from Turkey: New Insights on the Evolutionary History of Sheep
Source: PLoS One. 2013 Dec 11;8(12):e81952. doi: 10.1371/journal.pone.0081952 (PMC3859546; doi:10.1371/journal.pone.0081952)
Supplement: Table S5 — Sequences used to construct neighbor joining tree and mismatch distributions and to compute genetic diversities. (DOC) [file pone.0081952.s007.doc]

**Table S5. Sequences used to construct neighbor joining tree and mismatch distributions and to compute genetic diversities**

| **Accession** | **Sample name** | **Haplogroup** | **Analysis** | **References** |
| --- | --- | --- | --- | --- |
| AY091490 | Ovis vignei | Outgroup | NJ tree | Hiendleder et al., 2002 |
| AY091491 | Ovis vignei | Outgroup | NJ tree | Hiendleder et al., 2002 |
| AF242347 | Ovis ammon | Outgroup | NJ tree | Hiendleder et al., 2002 |
| AF242348 | Ovis ammon | Outgroup | NJ tree | Hiendleder et al., 2002 |
| HM236174 | Ovis aries cl122 | HPG A | NJ tree | Meadows et al., 2011 |
| HM236175 | Ovis aries r359 | HPG A | NJ tree | Meadows et al., 2011 |
| HM236176 | Ovis aries kk1 | HPG B | NJ tree | Meadows et al., 2011 |
| HM236177 | Ovis aries kk2 | HPG B | NJ tree | Meadows et al., 2011 |
| HM236178 | Ovis aries kk12 | HPG C | NJ tree | Meadows et al., 2011 |
| HM236179 | Ovis aries mk4 | HPG C | NJ tree | Meadows et al., 2011 |
| HM236180 | Ovis aries mk3 | HPG D | NJ tree | Meadows et al., 2011 |
| HM236181 | Ovis aries mk9 | HPG D | NJ tree | Meadows et al., 2011 |
| HM236182 | Ovis aries aw25 | HPG E | NJ tree | Meadows et al., 2011 |
| HM236183 | Ovis aries tj6 | HPG E | NJ tree | Meadows et al., 2011 |
| KF677024 | Ovis aries KRY_1 | HPG B | NJ tree | Present Study |
| KF677025 | Ovis aries KRY_3 | HPG E | NJ tree | Present Study |
| KF677026 | Ovis aries KRY_6 | HPG A | NJ tree | Present Study |
| KF677027 | Ovis aries KRY_7 | HPG C | NJ tree | Present Study |
| KF677028 | Ovis aries KRY_8 | HPG C | NJ tree | Present Study |
| KF677029 | Ovis aries KRY_11 | HPG A | NJ tree | Present Study |
| KF677030 | Ovis aries KRY_14 | HPG A | NJ tree | Present Study |
| KF677031 | Ovis aries KRY_25 | HPG B | NJ tree | Present Study |
| KF677032 | Ovis aries KRY_26 | HPG B | NJ tree | Present Study |
| KF677033 | Ovis aries KRY_27 | HPG A | NJ tree | Present Study |
| KF677034 | Ovis aries KRY_35 | HPG B | NJ tree | Present Study |
| KF677035 | Ovis aries KRY_38 | HPG A | NJ tree | Present Study |
| KF677036 | Ovis aries KRY_40 | HPG B | NJ tree | Present Study |
| KF677037 | Ovis aries KRY_44 | HPG C | NJ tree | Present Study |
| KF677038 | Ovis aries KRY_48 | HPG B | NJ tree | Present Study |
| KF677039 | Ovis aries AKK_1 | HPG B | NJ tree | Present Study |
| KF677040 | Ovis aries AKK_10 | HPG C | NJ tree | Present Study |
| KF677041 | Ovis aries AKK_12 | HPG C | NJ tree | Present Study |
| KF677042 | Ovis aries AKK_13 | HPG E | NJ tree | Present Study |
| KF677043 | Ovis aries AKK_14 | HPG B | NJ tree | Present Study |
| KF677044 | Ovis aries AKK_16 | HPG C | NJ tree | Present Study |
| KF677045 | Ovis aries AKK_19 | HPG A | NJ tree | Present Study |
| KF677046 | Ovis aries AKK_23 | HPG C | NJ tree | Present Study |
| KF677047 | Ovis aries AKK_24 | HPG A | NJ tree | Present Study |
| KF677048 | Ovis aries AKK_25 | HPG B | NJ tree | Present Study |
| KF677049 | Ovis aries AKK_26 | HPG B | NJ tree | Present Study |
| KF677050 | Ovis aries AKK_28 | HPG A | NJ tree | Present Study |
| KF677051 | Ovis aries AKK_30 | HPG A | NJ tree | Present Study |
| KF677052 | Ovis aries AKK_32 | HPG C | NJ tree | Present Study |
| KF677053 | Ovis aries AKK_34 | HPG C | NJ tree | Present Study |
| KF677054 | Ovis aries AKK_35 | HPG C | NJ tree | Present Study |
| KF677055 | Ovis aries AKK_38 | HPG E | NJ tree | Present Study |
| KF677056 | Ovis aries AKK_39 | HPG D | NJ tree | Present Study |
| KF677057 | Ovis aries AKK_45 | HPG E | NJ tree | Present Study |
| KF677058 | Ovis aries AKK_46 | HPG A | NJ tree | Present Study |
| KF677059 | Ovis aries AKK_47 | HPG A | NJ tree | Present Study |
| KF677060 | Ovis aries GOK_2 | HPG A | NJ tree | Present Study |
| KF677061 | Ovis aries GOK_8 | HPG B | NJ tree | Present Study |
| KF677062 | Ovis aries GOK_17 | HPG A | NJ tree | Present Study |
| KF677063 | Ovis aries GOK_22 | HPG A | NJ tree | Present Study |
| KF677064 | Ovis aries GOK_23 | HPG B | NJ tree | Present Study |
| KF677065 | Ovis aries GOK_27 | HPG A | NJ tree | Present Study |
| KF677066 | Ovis aries GOK_34 | HPG B | NJ tree | Present Study |
| KF677067 | Ovis aries GOK_38 | HPG B | NJ tree | Present Study |
| KF677068 | Ovis aries GOK_39 | HPG B | NJ tree | Present Study |
| KF677069 | Ovis aries GOK_41 | HPG A | NJ tree | Present Study |
| KF677070 | Ovis aries GOK_48 | HPG B | NJ tree | Present Study |
| KF677071 | Ovis aries DAG_1 | HPG C | NJ tree | Present Study |
| KF677072 | Ovis aries DAG_4 | HPG B | NJ tree | Present Study |
| KF677073 | Ovis aries DAG_8 | HPG B | NJ tree | Present Study |
| KF677074 | Ovis aries DAG_9 | HPG A | NJ tree | Present Study |
| KF677075 | Ovis aries DAG_10 | HPG C | NJ tree | Present Study |
| KF677076 | Ovis aries DAG_12 | HPG C | NJ tree | Present Study |
| KF677077 | Ovis aries DAG_21 | HPG A | NJ tree | Present Study |
| KF677078 | Ovis aries DAG_22 | HPG B | NJ tree | Present Study |
| KF677079 | Ovis aries DAG_24 | HPG B | NJ tree | Present Study |
| KF677080 | Ovis aries DAG_25 | HPG A | NJ tree | Present Study |
| KF677081 | Ovis aries DAG_32 | HPG B | NJ tree | Present Study |
| KF677082 | Ovis aries DAG_33 | HPG B | NJ tree | Present Study |
| KF677083 | Ovis aries DAG_35 | HPG C | NJ tree | Present Study |
| KF677084 | Ovis aries DAG_36 | HPG E | NJ tree | Present Study |
| KF677085 | Ovis aries DAG_39 | HPG C | NJ tree | Present Study |
| KF677086 | Ovis aries DAG_40 | HPG C | NJ tree | Present Study |
| KF677087 | Ovis aries DAG_44 | HPG C | NJ tree | Present Study |
| KF677088 | Ovis aries DAG_47 | HPG B | NJ tree | Present Study |
| KF677089 | Ovis aries DAG_50 | HPG A | NJ tree | Present Study |
| KF677090 | Ovis aries DAG_55 | HPG C | NJ tree | Present Study |
| KF677091 | Ovis aries DAG_56 | HPG A | NJ tree | Present Study |
| KF677092 | Ovis aries DAG_57 | HPG A | NJ tree | Present Study |
| KF677093 | Ovis aries IVE_2 | HPG B | NJ tree | Present Study |
| KF677094 | Ovis aries IVE_3 | HPG C | NJ tree | Present Study |
| KF677095 | Ovis aries IVE_7 | HPG A | NJ tree | Present Study |
| KF677096 | Ovis aries IVE_8 | HPG B | NJ tree | Present Study |
| KF677097 | Ovis aries IVE_9 | HPG B | NJ tree | Present Study |
| KF677098 | Ovis aries IVE_10 | HPG B | NJ tree | Present Study |
| KF677099 | Ovis aries IVE_14 | HPG C | NJ tree | Present Study |
| KF677100 | Ovis aries IVE_15 | HPG A | NJ tree | Present Study |
| KF677101 | Ovis aries IVE_19 | HPG C | NJ tree | Present Study |
| KF677102 | Ovis aries IVE_23 | HPG A | NJ tree | Present Study |
| KF677103 | Ovis aries IVE_25 | HPG C | NJ tree | Present Study |
| KF677104 | Ovis aries IVE_26 | HPG B | NJ tree | Present Study |
| KF677105 | Ovis aries IVE_28 | HPG C | NJ tree | Present Study |
| KF677106 | Ovis aries IVE_31 | HPG A | NJ tree | Present Study |
| KF677107 | Ovis aries IVE_32 | HPG C | NJ tree | Present Study |
| KF677108 | Ovis aries IVE_34 | HPG A | NJ tree | Present Study |
| KF677109 | Ovis aries IVE_36 | HPG B | NJ tree | Present Study |
| KF677110 | Ovis aries IVE_38 | HPG C | NJ tree | Present Study |
| KF677111 | Ovis aries IVE_41 | HPG C | NJ tree | Present Study |
| KF677112 | Ovis aries IVE_42 | HPG A | NJ tree | Present Study |
| KF677113 | Ovis aries IVE_44 | HPG A | NJ tree | Present Study |
| KF677114 | Ovis aries IVE_45 | HPG C | NJ tree | Present Study |
| KF677115 | Ovis aries IVE_47 | HPG C | NJ tree | Present Study |
| KF677116 | Ovis aries IVE_48 | HPG A | NJ tree | Present Study |
| KF677117 | Ovis aries IVE_49 | HPG C | NJ tree | Present Study |
| KF677118 | Ovis aries IVE_50 | HPG B | NJ tree | Present Study |
| KF677119 | Ovis aries HER_4 | HPG A | NJ tree | Present Study |
| KF677120 | Ovis aries HER_7 | HPG B | NJ tree | Present Study |
| KF677121 | Ovis aries HER_13 | HPG C | NJ tree | Present Study |
| KF677122 | Ovis aries HER_14 | HPG B | NJ tree | Present Study |
| KF677123 | Ovis aries HER_16 | HPG B | NJ tree | Present Study |
| KF677124 | Ovis aries HER_23 | HPG A | NJ tree | Present Study |
| KF677125 | Ovis aries HER_24 | HPG A | NJ tree | Present Study |
| KF677126 | Ovis aries HER_26 | HPG C | NJ tree | Present Study |
| KF677127 | Ovis aries HER_30 | HPG B | NJ tree | Present Study |
| KF677128 | Ovis aries HER_31 | HPG A | NJ tree | Present Study |
| KF677129 | Ovis aries HER_36 | HPG B | NJ tree | Present Study |
| KF677130 | Ovis aries HER_39 | HPG B | NJ tree | Present Study |
| KF677131 | Ovis aries HER_40 | HPG B | NJ tree | Present Study |
| KF677132 | Ovis aries HER_45 | HPG B | NJ tree | Present Study |
| KF677133 | Ovis aries HER_49 | HPG A | NJ tree | Present Study |
| KF677134 | Ovis aries KRG_1 | HPG C | NJ tree | Present Study |
| KF677135 | Ovis aries KRG_2 | HPG A | NJ tree | Present Study |
| KF677136 | Ovis aries KRG_4 | HPG B | NJ tree | Present Study |
| KF677137 | Ovis aries KRG_8 | HPG C | NJ tree | Present Study |
| KF677138 | Ovis aries KRG_9 | HPG B | NJ tree | Present Study |
| KF677139 | Ovis aries KRG_12 | HPG B | NJ tree | Present Study |
| KF677140 | Ovis aries KRG_13 | HPG A | NJ tree | Present Study |
| KF677141 | Ovis aries KRG_16 | HPG A | NJ tree | Present Study |
| KF677142 | Ovis aries KRG_17 | HPG E | NJ tree | Present Study |
| KF677143 | Ovis aries KRG_20 | HPG B | NJ tree | Present Study |
| KF677144 | Ovis aries KRG_21 | HPG E | NJ tree | Present Study |
| KF677145 | Ovis aries KRG_23 | HPG A | NJ tree | Present Study |
| KF677146 | Ovis aries KRG_24 | HPG A | NJ tree | Present Study |
| KF677147 | Ovis aries KRG_32 | HPG B | NJ tree | Present Study |
| KF677148 | Ovis aries KRG_38 | HPG B | NJ tree | Present Study |
| KF677149 | Ovis aries KRG_41 | HPG B | NJ tree | Present Study |
| KF677150 | Ovis aries KRG_44 | HPG B | NJ tree | Present Study |
| KF677151 | Ovis aries KRG_51 | HPG C | NJ tree | Present Study |
| KF677152 | Ovis aries KRG_53 | HPG A | NJ tree | Present Study |
| KF677153 | Ovis aries KRG_54 | HPG A | NJ tree | Present Study |
| KF677154 | Ovis aries CIC_1 | HPG A | NJ tree | Present Study |
| KF677155 | Ovis aries CIC_6 | HPG A | NJ tree | Present Study |
| KF677156 | Ovis aries CIC_7 | HPG B | NJ tree | Present Study |
| KF677157 | Ovis aries CIC_10 | HPG A | NJ tree | Present Study |
| KF677158 | Ovis aries CIC_16 | HPG C | NJ tree | Present Study |
| KF677159 | Ovis aries CIC_18 | HPG B | NJ tree | Present Study |
| KF677160 | Ovis aries CIC_22 | HPG B | NJ tree | Present Study |
| KF677161 | Ovis aries CIC_23 | HPG C | NJ tree | Present Study |
| KF677162 | Ovis aries CIC_24 | HPG C | NJ tree | Present Study |
| KF677163 | Ovis aries CIC_27 | HPG B | NJ tree | Present Study |
| KF677164 | Ovis aries CIC_29 | HPG A | NJ tree | Present Study |
| KF677165 | Ovis aries CIC_30 | HPG B | NJ tree | Present Study |
| KF677166 | Ovis aries CIC_31 | HPG B | NJ tree | Present Study |
| KF677167 | Ovis aries CIC_34 | HPG C | NJ tree | Present Study |
| KF677168 | Ovis aries CIC_35 | HPG B | NJ tree | Present Study |
| KF677169 | Ovis aries CIC_36 | HPG C | NJ tree | Present Study |
| KF677170 | Ovis aries SAK_5 | HPG B | NJ tree | Present Study |
| KF677171 | Ovis aries SAK_7 | HPG B | NJ tree | Present Study |
| KF677172 | Ovis aries SAK_8 | HPG B | NJ tree | Present Study |
| KF677173 | Ovis aries SAK_16 | HPG B | NJ tree | Present Study |
| KF677174 | Ovis aries SAK_17 | HPG C | NJ tree | Present Study |
| KF677175 | Ovis aries SAK_18 | HPG C | NJ tree | Present Study |
| KF677176 | Ovis aries SAK_21 | HPG B | NJ tree | Present Study |
| KF677177 | Ovis aries SAK_24 | HPG A | NJ tree | Present Study |
| KF677178 | Ovis aries SAK_29 | HPG B | NJ tree | Present Study |
| KF677179 | Ovis aries SAK_32 | HPG B | NJ tree | Present Study |
| KF677180 | Ovis aries SAK_35 | HPG B | NJ tree | Present Study |
| KF677181 | Ovis aries SAK_37 | HPG A | NJ tree | Present Study |
| KF677182 | Ovis aries SAK_39 | HPG B | NJ tree | Present Study |
| KF677183 | Ovis aries SAK_42 | HPG B | NJ tree | Present Study |
| KF677184 | Ovis aries SAK_43 | HPG B | NJ tree | Present Study |
| KF677185 | Ovis aries SAK_45 | HPG B | NJ tree | Present Study |
| KF677186 | Ovis aries SAK_47 | HPG C | NJ tree | Present Study |
| KF677187 | Ovis aries SAK_48 | HPG C | NJ tree | Present Study |
| KF677188 | Ovis aries SAK_49 | HPG B | NJ tree | Present Study |
| KF677189 | Ovis aries NOR_2 | HPG B | NJ tree | Present Study |
| KF677190 | Ovis aries NOR_4 | HPG A | NJ tree | Present Study |
| KF677191 | Ovis aries NOR_8 | HPG C | NJ tree | Present Study |
| KF677192 | Ovis aries NOR_12 | HPG A | NJ tree | Present Study |
| KF677193 | Ovis aries NOR_13 | HPG B | NJ tree | Present Study |
| KF677194 | Ovis aries NOR_16 | HPG C | NJ tree | Present Study |
| KF677195 | Ovis aries NOR_17 | HPG C | NJ tree | Present Study |
| KF677196 | Ovis aries NOR_18 | HPG C | NJ tree | Present Study |
| KF677197 | Ovis aries NOR_21 | HPG A | NJ tree | Present Study |
| KF677198 | Ovis aries NOR_22 | HPG C | NJ tree | Present Study |
| KF677199 | Ovis aries NOR_23 | HPG B | NJ tree | Present Study |
| KF677200 | Ovis aries NOR_24 | HPG C | NJ tree | Present Study |
| KF677201 | Ovis aries NOR_26 | HPG C | NJ tree | Present Study |
| KF677202 | Ovis aries NOR_27 | HPG C | NJ tree | Present Study |
| KF677203 | Ovis aries NOR_28 | HPG A | NJ tree | Present Study |
| KF677204 | Ovis aries NOR_29 | HPG B | NJ tree | Present Study |
| KF677205 | Ovis aries NOR_35 | HPG C | NJ tree | Present Study |
| KF677206 | Ovis aries NOR_40 | HPG A | NJ tree | Present Study |
| KF677207 | Ovis aries NOR_42 | HPG B | NJ tree | Present Study |
| KF677208 | Ovis aries NOR_43 | HPG C | NJ tree | Present Study |
| KF677209 | Ovis aries NOR_45 | HPG D | NJ tree | Present Study |
| KF677210 | Ovis aries NOR_46 | HPG C | NJ tree | Present Study |
| KF677211 | Ovis aries HEM_2 | HPG B | NJ tree | Present Study |
| KF677212 | Ovis aries HEM_3 | HPG A | NJ tree | Present Study |
| KF677213 | Ovis aries HEM_5 | HPG B | NJ tree | Present Study |
| KF677214 | Ovis aries HEM_7 | HPG B | NJ tree | Present Study |
| KF677215 | Ovis aries HEM_10 | HPG A | NJ tree | Present Study |
| KF677216 | Ovis aries HEM_14 | HPG A | NJ tree | Present Study |
| KF677217 | Ovis aries HEM_17 | HPG B | NJ tree | Present Study |
| KF677218 | Ovis aries HEM_21 | HPG C | NJ tree | Present Study |
| KF677219 | Ovis aries HEM_23 | HPG A | NJ tree | Present Study |
| KF677220 | Ovis aries HEM_24 | HPG A | NJ tree | Present Study |
| KF677221 | Ovis aries HEM_25 | HPG A | NJ tree | Present Study |
| KF677222 | Ovis aries HEM_26 | HPG B | NJ tree | Present Study |
| KF677223 | Ovis aries HEM_32 | HPG C | NJ tree | Present Study |
| KF677224 | Ovis aries HEM_39 | HPG A | NJ tree | Present Study |
| KF677225 | Ovis aries HEM_41 | HPG A | NJ tree | Present Study |
| KF677226 | Ovis aries HEM_42 | HPG B | NJ tree | Present Study |
| KF677227 | Ovis aries HEM_43 | HPG B | NJ tree | Present Study |
| KF677228 | Ovis aries HEM_44 | HPG C | NJ tree | Present Study |
| KF677229 | Ovis aries KIV_4 | HPG B | NJ tree | Present Study |
| KF677230 | Ovis aries KIV_9 | HPG B | NJ tree | Present Study |
| KF677231 | Ovis aries KIV_15 | HPG C | NJ tree | Present Study |
| KF677232 | Ovis aries KIV_18 | HPG B | NJ tree | Present Study |
| KF677233 | Ovis aries KIV_29 | HPG B | NJ tree | Present Study |
| KF677234 | Ovis aries KIV_40 | HPG B | NJ tree | Present Study |
| KF677235 | Ovis aries KIV_43 | HPG C | NJ tree | Present Study |
| KF677236 | Ovis aries KIV_47 | HPG B | NJ tree | Present Study |
| KF677237 | Ovis aries KIV_51 | HPG B | NJ tree | Present Study |
| KF677238 | Ovis aries MRK_1 | HPG A | NJ tree | Present Study |
| KF677239 | Ovis aries MRK_6 | HPG A | NJ tree | Present Study |
| KF677240 | Ovis aries MRK_8 | HPG C | NJ tree | Present Study |
| KF677241 | Ovis aries MRK_9 | HPG C | NJ tree | Present Study |
| KF677242 | Ovis aries MRK_12 | HPG B | NJ tree | Present Study |
| KF677243 | Ovis aries MRK_14 | HPG E | NJ tree | Present Study |
| KF677244 | Ovis aries MRK_17 | HPG B | NJ tree | Present Study |
| KF677245 | Ovis aries MRK_18 | HPG C | NJ tree | Present Study |
| KF677246 | Ovis aries MRK_19 | HPG A | NJ tree | Present Study |
| KF677247 | Ovis aries MRK_23 | HPG A | NJ tree | Present Study |
| KF677248 | Ovis aries MRK_24 | HPG E | NJ tree | Present Study |
| KF677249 | Ovis aries MRK_28 | HPG C | NJ tree | Present Study |
| KF677250 | Ovis aries MRK_29 | HPG E | NJ tree | Present Study |
| KF677251 | Ovis aries MRK_30 | HPG C | NJ tree | Present Study |
| KF677252 | Ovis aries MRK_31 | HPG C | NJ tree | Present Study |
| KF677253 | Ovis aries MRK_32 | HPG A | NJ tree | Present Study |
| KF677254 | Ovis aries MRK_33 | HPG B | NJ tree | Present Study |
| KF677255 | Ovis aries MRK_37 | HPG E | NJ tree | Present Study |
| KF677256 | Ovis aries MRK_38 | HPG A | NJ tree | Present Study |
| KF677257 | Ovis aries MRK_41 | HPG C | NJ tree | Present Study |
| KF677258 | Ovis aries MRK_42 | HPG A | NJ tree | Present Study |
| KF677259 | Ovis aries MRK_43 | HPG C | NJ tree | Present Study |
| KF677260 | Ovis aries MRK_44 | HPG A | NJ tree | Present Study |
| KF677261 | Ovis aries MRK_46 | HPG C | NJ tree | Present Study |
| KF677262 | Ovis aries MRK_47 | HPG A | NJ tree | Present Study |
| KF677263 | Ovis aries MRK_48 | HPG C | NJ tree | Present Study |
| KF677264 | Ovis gmelinii anatolica OGA_1 | TypeX | NJ tree | Present Study |
| KF677265 | Ovis gmelinii anatolica OGA_2 | TypeX | NJ tree | Present Study |
| KF677266 | Ovis gmelinii anatolica OGA_3 | TypeX | NJ tree | Present Study |
| KF677267 | Ovis gmelinii anatolica OGA_4 | HPG A | NJ tree | Present Study |
| KF677268 | Ovis gmelinii anatolica OGA_5 | HPG A | NJ tree | Present Study |
| KF677269 | Ovis gmelinii anatolica OGA_6 | HPG A | NJ tree | Present Study |
| KF677270 | Ovis gmelinii anatolica OGA_7 | HPG A | NJ tree | Present Study |
| KF677271 | Ovis gmelinii anatolica OGA_8 | TypeX | NJ tree | Present Study |
| KF677272 | Ovis gmelinii anatolica OGA_9 | HPG A | NJ tree | Present Study |
| KF677273 | Ovis gmelinii anatolica OGA_10 | HPG A | NJ tree | Present Study |
| KF677274 | Ovis gmelinii anatolica OGA_11 | HPG A | NJ tree | Present Study |
| KF677275 | Ovis gmelinii anatolica OGA_12 | TypeX | NJ tree | Present Study |
| KF677276 | Ovis gmelinii anatolica OGA_13 | HPG A | NJ tree | Present Study |
| KF677277 | Ovis gmelinii anatolica OGA_14 | TypeX | NJ tree | Present Study |
| KF677278 | Ovis gmelinii anatolica OGA_15 | HPG A | NJ tree | Present Study |
| KF677279 | Ovis gmelinii anatolica OGA_16 | HPG A | NJ tree | Present Study |
| KF677280 | Ovis gmelinii anatolica OGA_17 | HPG A | NJ tree | Present Study |
| KF677281 | Ovis gmelinii anatolica OGA_18 | HPG A | NJ tree | Present Study |
| KF677282 | Ovis gmelinii anatolica OGA_19 | HPG A | NJ tree | Present Study |
| KF677283 | Ovis gmelinii anatolica OGA_20 | HPG A | NJ tree | Present Study |
| KF677284 | Ovis gmelinii anatolica OGA_21 | TypeX | NJ tree | Present Study |
| KF677285 | Ovis gmelinii anatolica OGA_22 | HPG A | NJ tree | Present Study |
| KF677286 | Ovis gmelinii anatolica OGA_23 | HPG A | NJ tree | Present Study |
| KF677287 | Ovis gmelinii anatolica OGA_24 | HPG A | NJ tree | Present Study |
| KF677288 | Ovis gmelinii anatolica OGA_25 | TypeX | NJ tree | Present Study |
| KF677289 | Ovis gmelinii anatolica OGA_26 | HPG A | NJ tree | Present Study |
| KF677290 | Ovis gmelinii anatolica OGA_27 | HPG A | NJ tree | Present Study |
| KF677291 | Ovis gmelinii anatolica OGA_28 | HPG A | NJ tree | Present Study |
| KF677292 | Ovis gmelinii anatolica OGA_29 | HPG A | NJ tree | Present Study |
| KF677293 | Ovis gmelinii anatolica OGA_30 | HPG A | NJ tree | Present Study |
| KF677024 | Ovis aries KRY_1 | HPG B | NJ tree | Present Study |
| HM236184 | Ovis musimon h1 | HPG B | Dxy calculation | Meadows et al., 2011 |
| HM236185 | Ovis musimon h2 | HPG B | Dxy calculation | Meadows et al., 2011 |
| AY091487 | Ovis musimon | HPG B | Dxy calculation | Hiendleder et al., 2002 |
| AY091488 | Ovis musimon | HPG B | Dxy calculation | Hiendleder et al., 2002 |
| AF039579 | Ovis musimon | HPG B | Dxy calculation | Hiendleder et al., 1998 |
| HM236182 | Ovis aries aw25 | HPG E | Mismatch distribution (E2+E3) | Meadows et al., 2011 |
| HM236183 | Ovis aries tj6 | HPG E | Mismatch distribution (E2+E3) | Meadows et al., 2011 |
| AY829385 | Ovis aries HSK018 | HPG E | Mismatch distribution (E2+E3) | Guo et al., 2005 |
| AY829404 | Ovis aries MG024 | HPG E | Mismatch distribution (E2+E3) | Guo et al., 2005 |
| DQ097468 | Ovis aries KAR15 | HPG E | Mismatch distribution (E3) | Pedrosa et al., 2005 |
| HM042760 | Ovis aries TR0005 | HPG E | Mismatch distribution (E3) | Koban et al., Unpublished |
| HM042761 | Ovis aries TR0006 | HPG E | Mismatch distribution (E3) | Koban et al., Unpublished |
| HM042785 | Ovis aries TR0030 | HPG E | Mismatch distribution (E3) | Koban et al., Unpublished |
| HM042838 | Ovis aries TR0083 | HPG E | Mismatch distribution (E3) | Koban et al., Unpublished |
