# Supplementary material for: Mitochondrial DNA Diversity of Modern, Ancient and Wild Sheep (Ovis gmelinii anatolica) from Turkey: New Insights on the Evolutionary History of Sheep
Source: PLoS One. 2013 Dec 11;8(12):e81952. doi: 10.1371/journal.pone.0081952 (PMC3859546; doi:10.1371/journal.pone.0081952)
Supplement: Table S6 — aDNA sample codes and their corresponding archaeological codes. (DOC) [file pone.0081952.s008.doc]

**Table S6. aDNA sample codes and their corresponding archaeological codes**

| **Sample code used in the article** | **Oylum Höyük archaeological code** |
| --- | --- |
| OY003-1 | OY 11/809, K22, 141#15 |
| OY018-1 | OY 11/814, K22, 141#17 |
| OY020-1 | OY 11/804, K22, 141#13 |
| OY024-1 | OY 11/394 a, K22, 141#11 |
| OY025-1 | OY 11/623, J21, 155#21 |
| OY065-2 | OY 09/1472, J22, 131#8 |
| OY010-1 | OY 11/394 c, K22, 141#11 |
| OY042-2 | OY 11/1330, K22, 146#3 |
| OY027-1 | OY 11/394 b, K22, 141#11 |
| OY134-2 | OY 11/864, K22, 141#20 |
| OY086-2 | OY 11/123, K21, 123#2 |
| OY130-2 | OY 11/1353 a, K22, 146#4 |
| OY044-2 | OY 11/1353 b, K22, 146#4 |
| OY133-2 | OY 12/3007, K21, 146#1 |
| OY105-2 | OY 12/1565, L21, 123#4 |
| OY067-2 | OY 09/1657, L22, 76#2 |
| OY070-2 | OY 11/1042, K21, 88#1 |
| OY061-2 | OY 10/1430, M22, 72#5 |
| OY059-2 | OY 10/1448, M22, 72#8 |
| OY090-2 | OY 10/798, M22, 61#7 |
| OY123-2 | OY 12/1504, L21, 61#4 |
| OY072-2 | OY 09/968, M21, 27#5 |
| OY019-2 | OY 12/3695, M23, 20#6 |
| OY078-2 | OY 12/4189a, L23, 25#1 |
| OY082-2 | OY 12/4713, K23, 31#1 |
| OY110-2 | OY 12/4687, K23, 27#1 |
| OY025-2 | OY 12/4548, K23, 13#1 |
| OY142-2 | OY 12/4069, L23, 16#1 |
| OY089-2 | OY 10/730, M22, 22#14 |
| OY091-2 | OY 09/207, L22, 24#1 |
| OY081-2 | OY 12/4189b, L23, 25#1 |
| OY138-2 | OY 12/4282, L23, 36#1 |
| OY021-2 | OY 12/4047, L23, 10#2 |
