# Supplementary material for: Mitochondrial DNA Diversity of Modern, Ancient and Wild Sheep (Ovis gmelinii anatolica) from Turkey: New Insights on the Evolutionary History of Sheep
Source: PLoS One. 2013 Dec 11;8(12):e81952. doi: 10.1371/journal.pone.0081952 (PMC3859546; doi:10.1371/journal.pone.0081952)
Supplement: Table S7 — The average number of nucleotide differences per site (Dxy) among the haplogroups of domestic sheep, O. g. anatolica and O. g. musimon. (DOC) [file pone.0081952.s009.doc]

**Table S7. The average number of nucleotide differences per site (Dxy) among the haplogroups of domestic sheep, *O. g.* *anatolica* and *O. g. musimon***

|  | **HPGA** | **HPGB** | **HPGC** | **HPGD** | **HPGE** |
| --- | --- | --- | --- | --- | --- |
| **HPG B** | 0.0328 |  |  |  |  |
| **HPG C** | 0.0368 | 0.0359 |  |  |  |
| **HPG D** | 0.0335 | 0.0334 | 0.0342 |  |  |
| **HPG E** | 0.0331 | 0.0375 | 0.0153 | 0.0345 |  |
| ***O. g. anatolica* X** | 0.0381 | 0.0413 | 0.0149 | 0.0403 | 0.0229 |
| ***O. g. anatolica* A** | 0.0032 |  |  |  |  |
| ***O. g. musimon*** |  | 0.0075 |  |  |  |
