# Supplementary material for: Mitochondrial DNA Diversity of Modern, Ancient and Wild Sheep (Ovis gmelinii anatolica) from Turkey: New Insights on the Evolutionary History of Sheep
Source: PLoS One. 2013 Dec 11;8(12):e81952. doi: 10.1371/journal.pone.0081952 (PMC3859546; doi:10.1371/journal.pone.0081952)
Supplement: Table S8 — Mean mismatch values, Fu's FS statistics and Tajima's D statistics of domestic haplogroups. (DOC) [file pone.0081952.s010.doc]

**Table S8. Mean mismatch values, Fu’s F_S_ statistics and Tajima’s *D* statistics of domestic haplogroups**

|  | Mean mismatch values | Fu’s F_S_ statistics | Tajima’s *D* statistics |
| --- | --- | --- | --- |
| HPG A | 3.759 | -26.0*** | -2.39*** |
| HPG B | 5.311 | -25.4*** | -2.32*** |
| HPG C | 2.959 | -26.5*** | -2.4*** |
| HPG E1 | 5.673 | -2.4* | -0.95*** |
| HPG E2 | 5.962 | -5.6*** | -1.2*** |
| HPG E3 | 5.463 | -9.7*** | -1.34*** |

*p<0.05, ***p<0.001
