# Supplementary material for: Mitochondrial DNA Diversity of Modern, Ancient and Wild Sheep (Ovis gmelinii anatolica) from Turkey: New Insights on the Evolutionary History of Sheep
Source: PLoS One. 2013 Dec 11;8(12):e81952. doi: 10.1371/journal.pone.0081952 (PMC3859546; doi:10.1371/journal.pone.0081952)
Supplement: Text S1 — Identification of ancient sheep samples. (DOC) [file pone.0081952.s011.doc]

**Text S1. Identification of ancient sheep samples**

The separation of sheep from goat was based on criteria published in archaeozoological literature. Only three skeletal elements were chosen: metacarpal, metatarsal and mandible with teeth. This limited selection was applied to increase the reliability of identification based on the fact that multiple criteria are described for these elements while for metacarpal and metatarsal additional osteometrical criteria are well established. Metacarpals and metatarsals were separated on 4 morphological criteria applicable on the distal epiphyses and distal shaft (distal shaft for metatarsal) as described by Boessneck [1] and evaluated by Zeder and Lapham [2]. Confirmation of the morphological criteria was done by using two osteometric methods, one described by Boessneck [1] and the other by Payne [3]. The separation of the mandibles was based on the work published by Payne [4] and Halstead et al. [5]. In addition, the discussion/evaluation of these criteria by Gillis et al. [6] was consulted. In order to identify a bone as “sheep” and to choose it for a DNA experiment, at least three of these criteria had to be definitely met and positively confirmed as “sheep” with no criterion on the same bone being scored as having “goat” characteristics.

**References:**

1. Boessneck J (1969) Osteological differences between sheep (Ovis aries Linné) and goats (Capra hircus Linné). In: Brothwell D, Higgs E, editors. Science in Archaeology, London, UK: Thames and Hudson. pp. 331–358.

2. Zeder MA, Lapham, HA (2010) Assessing the reliability of criteria used to identify postcranial bones in sheep, Ovis and goats, Capra. Journal of Archaeological Science, 37: 2887-2905.

3. Payne S (1969) A metrical distinction between sheep and goat metacarpals. In: Ucko PJ, Dimbleby GW, editors. The Domestication and Exploitation of Plants and Animals, London, UK: Duckworth Pub. pp. 295-305.

4. Payne S (1985) Morphological distinctions between the mandibular teeth of young sheep, Ovis, and goats, Capra. Journal of Archaeological Science 12: 139-147.

5. Halstead P, Collins P, Isaakidou V (2002) Sorting the sheep from the goats: morphological distinctions between the mandibles and mandibular teeth of adult Ovis and Capra. Journal of Archaeological Science 29: 545-553.

6. Gillis R, Chaix L, Vigne JD (2010) An assessment of morphological criteria for discriminating sheep and goat mandibles on a large prehistoric archaeological assemblage (Kerma, Sudan) Journal of Archaeological Science 38: 2324-2339.
