# Supplementary material for: Mitochondrial DNA Diversity of Modern, Ancient and Wild Sheep (Ovis gmelinii anatolica) from Turkey: New Insights on the Evolutionary History of Sheep
Source: PLoS One. 2013 Dec 11;8(12):e81952. doi: 10.1371/journal.pone.0081952 (PMC3859546; doi:10.1371/journal.pone.0081952)
Supplement: Text S2 — Study site for aDNA. (DOC) [file pone.0081952.s012.doc]

**Text S2. Study site for aDNA**

Oylum Höyük, within the borders of Kilis, is located between provinces of Gaziantep and Kilis (Turkey) and 55 km north of Aleppo (Syria). The mound is composed of two hills, with the heights of 22 m and 37 m, connected by a neck and it covers an area of approximately 17 hectares [1]. A surface survey around the region was conducted by Prof. Dr. Engin Özgen in 1985 and the excavations have started two years later, in 1987 [2].

The stratigraphy of unearthed layers of the mound until the time of the reference study [1] is as follows:

1. Byzantine Period
2. Roman Period
3. Hellenistic Period
4. Iron Age
5. Late Bronze Age
6. Middle Bronze Age
7. Early Bronze Age III-IV
8. Early Bronze Age I-II
9. Early Bronze Age I-Late Uruk Transition Period
10. Late Uruk Period

It is reported that the mound was continuously inhabited, at least between these periods.

**Information on aDNA samples and the measures taken for preventing contamination**

Teeth attached to the mandibles and metapodia were used in the study. One of the most important reasons, to use teeth for aDNA studies, is; they are highly useful in differentiating sheep samples from goat. Moreover, as these types of samples are poor in terms of nutrition they are usually found intact or semi-intact presenting no or few butchery marks. The samples were collected between the years 2008-2012 by archaeologists and stored at the repository. After their examination for identification the samples were brought to our aDNA laboratory and have been kept at +4^o^C. All of the samples are unburned.

Manipulation of samples, DNA extraction/pre-PCR and PCR together with post-PCR steps were performed in physically separated rooms. The necessary precautions were taken to prevent possible contaminations from modern DNA. Whenever the samples were manipulated, the laboratory personnel used single-use facial masks, two pairs of gloves, full body laboratory coat and slippers. The surfaces and equipment were treated with bleach and also UV radiated prior to and after performing the experiments. After PCR step, the samples were not handled for another extraction in the same day. The DNA sequences we obtained were subjected to BLAST incorporated in the ChromasPro version 1.7.5 (<http://technelysium.com.au/>) to ensure that all of them were assigned to *Ovis aries* with a minimum query coverage of 99% and identity of 97%.

**References:**

1. Erdem Dağ Ö (2004) Orta Tuç Çağı Güneydoğu Anadolu ve Kuzey Suriye ölü gömme gelenekleri ışığında Oylum Höyük Mezarları [Oylum Hoyuk burial sites in the light of Middle Bronze Age burial customs in Southeastern Anatolia and Northern Syria]. Master Thesis, Hacettepe University, Ankara, Turkey.
2. Özgen E (1986) Gaziantep Kilis bölgesi yüzey araştırmaları [Surface surveys in Gaziantep Kilis region]. 4th Research Results Symposium of General Directorate of Monuments and Museums, Turkish Ministry of Culture and Tourism, Ankara, Turkey.
